# *In Silico* Models for Dynamic Connected Cell Cultures Mimicking Hepatocyte-Endothelial Cell-Adipocyte Interaction Circle

**DOI:** 10.1371/journal.pone.0111946

**Published:** 2014-12-15

**Authors:** Chiara Andreoni, Gianni Orsi, Carmelo De Maria, Francesca Montemurro, Giovanni Vozzi

**Affiliations:** 1 Research Center “E. Piaggio”, University of Pisa, Pisa, Italy; 2 Department of Information Engineering, University of Pisa, Pisa, Italy; Glasgow University, United Kingdom

## Abstract

The biochemistry of a system made up of three kinds of cell is virtually impossible to work out without the use of *in silico* models. Here, we deal with homeostatic balance phenomena from a metabolic point of view and we present a new computational model merging three single-cell models, already available from our research group: the first model reproduced the metabolic behaviour of a hepatocyte, the second one represented an endothelial cell, and the third one described an adipocyte. Multiple interconnections were created among these three models in order to mimic the main physiological interactions that are known for the examined cell phenotypes. The ultimate aim was to recreate the accomplishment of the homeostatic balance as it was observed for an *in vitro* connected three-culture system concerning glucose and lipid metabolism in the presence of the medium flow. The whole model was based on a modular approach and on a set of nonlinear differential equations implemented in Simulink, applying Michaelis-Menten kinetic laws and some energy balance considerations to the studied metabolic pathways. Our *in silico* model was then validated against experimental datasets coming from literature about the cited *in vitro* model. The agreement between simulated and experimental results was good and the behaviour of the connected culture system was reproduced through an adequate parameter evaluation. The developed model may help other researchers to investigate further about integrated metabolism and the regulation mechanisms underlying the physiological homeostasis.

## Introduction

Over the past decades, the advent of high-throughput biotechnologies, such as genomics and proteomics, has allowed a better understanding of the cellular and molecular networks on which life is based. This fact, in turn, has forced a review of the concept of a cell as a mere collection of components to be treated separately [1]. It has become increasingly clear that signalling pathways interact with one another and the final biological response is shaped by these interactions. The resulting network of interactions is quite complex and may have properties that are non-intuitive, which are often dependent on subtle timing relations and competitions among regulators [2]. Researchers have thus moved towards an integrative approach aiming to unveil the system properties that can emerge from the complex interaction of basic elements [1]. Reproducing functional tissues *ex vivo*, for example, requires an understanding not only of the behaviour of individual cells, but also of how global shape and function arise from local cellular interactions [3].

Systems biology applies quantitative, mechanistic modelling to study genetic networks, signal transduction pathways and metabolic networks with the aim to yield a more global, in-depth and integrated understanding of biological systems. From a systems biology perspective, for example, all living organisms share a notable feature, which is afforded by the interconnection and co-execution of different functionalities: a high level of robustness against external and internal perturbations [4].

System level understanding of complex processes is common in engineering disciplines and relies heavily on mathematical models, informatics and methods from systems theory. The biological field is indeed facing an increased use of mathematical models and computer simulations; therefore, this approach is often referred to as “*in silico* biology”. The introduction of computational methods and instruments to complement the traditional experimental ones accelerates the generation of new hypotheses, and the research validation cycle [5]. The mathematical modelling of complex biological systems is of iterative and multidisciplinary kind: hypotheses emerging from *in silico* analyses are validated by experimenters, and the results are used to update the computational model exploited, thus involving various scientific fields [1].

*In silico* biology also offers many advantages over *in vivo* and *in vitro* experiments: quick predictions of phenomena of interest, a great reduction of production costs, no ethical problems (no animal needed, in line with the “3R rule” [6]), user-friendly interfaces for an easy and intuitive approach, the damping of data dispersion, which is typical of experimentally obtained parameters, and a broad spectrum of applications (e.g., drug testing field).

Literature offers many attempts to model and simulate molecular processes, ranging from genetic regulatory networks to metabolic pathways, and presents several software packages, such as MIST [7], GEPASI [8], SCAMP [9], JARNAC [10], METAMODEL [11], E-CELL SE [12], BIOSPICE [13]. Most of these packages have been developed for the quantitative simulation of biochemical metabolic pathways and are based on the numerical integration of rate equations, focusing only on metabolic aspects, without considering the environment in which cells are immersed. Our work group, instead, has defined a different collection of software platforms [14] [15] [16] [17] [18] with the precise purpose to reproduce physical-chemical and intercellular interactions, to which cells living in a physiological or an *in vitro* context are typically subjected, and the effects of these stimuli on metabolism. Starting from online metabolic pathways databases, such as the Kyoto Encyclopedia of Genes and Genomes (KEGG), and relating them to the systems theory language, our group developed HEMETβ (HEpatocyte METabolism mathematical model β) [16], ENMET (ENdothelial METabolism mathematical model) [17] and ADMET (ADipocyte METabolism mathematical model) [18], three virtual cell models reproducing the hepatocyte, the endothelial cell and the adipocyte metabolism, respectively. A subsequent model, CREPE (mathematical model for CRoss-talking of Endothelial cells and hePatocytE metabolism) [19], was originated through the suitable merging of HEMETβ and ENMET in order to simulate the metabolic behaviour of static hepatocyte and endothelial cell co-cultures. The different platforms all derived from the same previous and stoichiometric model, HEMET (HEpatocyte METabolism mathematical model) [14]. All these models, implemented in Simulink (The MathWorks, Inc.), were able to mimic the dynamic behaviour of cell monocultures or co-cultures as a function of variations in metabolite concentrations, using single enzymatic reactions as basic functional blocks for all the metabolic pathways involved. The models were characterized by a modular structure that enabled users to explore, add/subtract and modify single modules easily, managing models through user-friendly interfaces. The Michaelis-Menten approach in almost-steady state regime was chosen for the mathematical modelling of enzymatic reactions, so defining basic blocks to duplicate and connect as needed in order to form complex reaction networks as the glycolytic or the aminoacidic ones are. However, the cited models represented only cell monoculture metabolic profile or the behaviour of static cell co-cultures. None of them was able to simulate the dynamic and mutual inter-change of metabolites and the accomplishment of homeostatic balance, which are widespread physiological phenomena. We then decided to turn attention to these noteworthy system properties.

*In vivo*, many physiological and pathophysiological processes involve a great network of signalling molecules, through which cells belonging to different tissues can communicate and cooperate in spite of physical distance [20]. The bodily fluids flowing through the spread vascular network are then essential for this interaction to be possible. In metabolic regulation, for instance, liver, adipose tissue, pancreas and muscle cooperate through several biochemical pathways triggered by hormones and small molecules, providing the body with the right amount of energy it needs, and storing energy substrates (i.e., homeostatic balance) [21]. This balance is usually deranged in obese and diabetic patients or in metabolic syndrome disease [22].

More simple *in vitro* or *in silico* models are needed to gain insight into these complex systems and to counteract pathological states with effective therapies. In a simplified model of energy substrate metabolism in the visceral region, three main tissues have to be considered as the first basic elements to reproduce: hepatic, adipose and endothelial tissues [23]. The liver has a central role because of its multiple anabolic and catabolic functions processing all energy substrates (fats, sugars and proteins). Adipose tissue is not only a fat storage depot, but it is also particularly sensitive to the overall nutritional status in the body, informing other organs about that. Endothelial cells act as modulators of molecular signalling: indeed, metabolites are transported along the vascular network, which connects different organs. An *in vitro* model of this three-tissue system already exists concerning the glucose and lipid metabolism, the aminoacidic degradation and the main synthetic functions [21] [24]. It is made up of a multicompartmental modular bioreactor (MCmB) [26] consisting of interconnected chambers, each one housing a specific tissue of interest. Each module can be addressed and interrogated separately and different cell types can be added stepwise to the system. The culture medium flow links different chambers much as the bloodstream connects different organs in the body: this system is indeed referred to as a “dynamic connected culture system”. The authors of studies [21] and [24] exploited MCmB for the reproduction of a downscaled *in vitro* human visceral region to study cross-talking phenomena and their effect on metabolic regulation. They showed this culture system to be suitable for baseline studies with cell monocultures [23] and for upgraded analysis with cultures of two or three cell types [21] [24]. Despite its simplicity, the overall system and monocultures were able to reproduce several characteristics of *in vivo* glucose and lipid metabolism and of homeostatic mechanisms.

In this paper, we introduce a new computational multi-scale model that merge the previous three single-cell models (HEMETβ, ENMET and ADMET) with adequate interconnections, relating metabolic regulation to molecular biochemical mechanisms. We aimed to reproduce the metabolic behaviour of three distinct cell culture systems only connected by the medium fluid flow (dynamic 3-way connected cell culture system): we conceived the final *in silico* model as a coexistence and functional integration of the three different metabolic profiles considered, attempting to recreate the homeostatic balance observed *in vitro*. At first, each cell type was modelled as a standalone entity, with its own proliferation rate and specific metabolic pathways. The standalone models were then “connected” by modelling molecular interactions among them through metabolite uptake/release phenomena, and different kinds of cellular connections, thus generating a 3-way (Hepatic-Endothelial-Adipose) model. Then, the model was validated against available experimental data coming from literature datasets about the dynamic *in vitro* model described above. Available data concerned only extracellular species in the culture medium besides measurements of cell population growth.

## Materials and Methods

### 1. *In Silico* Model Basic Structure

Our model implements metabolic networks using nonlinear differential equations and systems theory approach, and linking biochemical pathways only to enzymatic reactions and metabolite inter-change. The presented model was created following the basic structure and design principles of previously developed models like HEMETβ [16], ENMET [17], ADMET [18] and CREPE [19]: they all involved Michaelis-Menten kinetics for reversible or irreversible reactions and for the enzymatic inhibition model, and the definition of energy constraints, such as availability of ATP or other high-energy molecules. Cell populations were assumed as homogeneous populations whose behaviour could thus be described by an average cell. In particular, we used a dynamic mathematical model with lumped parameters: the usual assumption was made that each compartment (i.e., the medium and the cell) was a lumped phase (i.e., concentrations are constant throughout the compartment). Conceptually, compartments correspond to cellular structures, such as organelles, pooled biochemical components or the cell environment, all of which are characterized by a spatial dimension. However, the ordinary differential equations actually represented distinct pooled concentrations at a single point and did not describe physical dimensions and therefore cell geometry [25]: time was the only independent variable of our system. Sidoli *et al.* [25] explain that mathematical models can be classified as structured or unstructured, segregated or unsegregated, and deterministic or stochastic. Firstly, a structured mathematical model includes a detailed description of the intracellular processes in either the physical or the biochemical sense, whereas these processes are only partially considered in unstructured models. In structured models, kinetic or stoichiometric equations are used to describe the intracellular reactions. This kind of models provides the advantages of flexibility and detail, but it has the drawback of obtaining data for parameter determination and model analysis with respect to the large number of equations involved. Secondly, an unsegregated mathematical model assumes an average cell so that the cell population can be considered homogeneous, without taking into account the differences conferred by the cell age, size, growth rate and metabolic state. Finally, deterministic models assume that the cells are not subject to random variability. Stochastic models capture cellular functions using probability distributions, thus taking into account randomness in the process. Therefore, according to these definitions, the mathematical model we used can be classified as structured, unsegregated, and deterministic.

Chemical stimuli (e.g., medium culture substances and nutrient availability) and physical ones (e.g., the presence of flow and, only for endothelial cells, a non-zero shear-stress value) were integrated in quantitative terms to the complex metabolic interconnected blocks chains. The modular layout, also in agreement with the corresponding *in vitro* model [24], and the user-friendly interface were conserved. Most of the involved parameters were considered time-invariant. In the model and during the validation phase, only temporal variations were evaluated for the metabolite concentrations: temporal scales for the experiments to be completed (hours and days) were much longer than typical metabolite transport times (generally minutes) through tissues. Metabolic pathways to implement were defined following online databases such as KEGG [27], BRaunschweig ENzyme DAtabase (BRENDA) [28] and consolidated biochemistry.

As hinted before, the basic blocks derived from Michaelis-Menten kinetic model for irreversible enzymatic reactions:
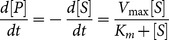
(1)where 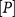 (mM) is the product concentration, 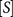 (mM) is the substrate concentration, and 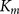 (mM) and 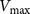 (mMs^−1^) are the Michaelis-Menten constant and the maximum catalysis rate of enzyme, respectively.

For reversible reactions, the Michaelis-Menten equation we referred to is:
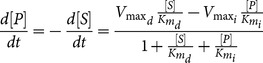
(2)where 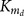 (mM) and 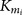 (mM) are the reaction Michaelis–Menten constants for direct and inverse reaction, respectively, and 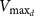 (mMs^−1^) and 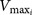 (mMs^−1^) are the maximum catalysis rate that can be reached in direct and inverse reaction, respectively. If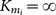, i.e. product release step is irreversible, Equation (2) becomes equal to Equation (1).

Competitive enzymatic inhibition was introduced in the model by Equation (3):
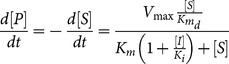
(3)where 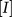 (mM) represents competitive inhibitor concentration and 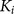 (mM) is the inhibition constant. As in the previous case, if 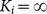, i.e. inhibitor is never bound to enzyme, Equation (3) becomes Equation (1).

In general, metabolic pathways involve reactions with multiple substrates and/or products and other equations have to be defined. Supposing to deal with an 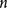
substrates-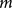
products reaction in which all reactants are taken up at the same instant, we can write more general equations. Therefore, equations (1) and (2) became respectively Equation (4) and (5):
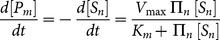
(4)




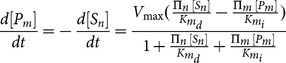
(5)where 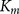, 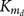 and 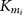 now are the mean of the corresponding 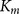, 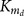 and 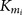 of each component.

In this model, the maximum rates in direct and inverse reactions are supposed equal (in accordance with their similar order of magnitude) and these parameters include correction factors to count other non-instantaneous processes, such as gene expression, that slow down global processes [16]. It is noteworthy that values for kinetic parameters, such as 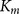 and 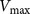, can be found in enzyme data bank (BRENDA), as we will see later: inevitably, this procedure introduces some uncertainty level in the model, due to non-standard experimental protocol and measurement units with which values are obtained [29] [30]. For most processes, indeed, kinetic parameters are not directly accessible *in vivo* and existing biochemical data usually originate from different experimental settings, cell types and state of cells.

In accordance with systems theory approach, it is possible to reproduce entire metabolic pathways as series of ODEs (Ordinary Differential Equations), in which metabolite concentrations are state variables and equations (4–5) become state equations. Considering these equations as basic Simulink blocks, according to block diagram algebra rules and linking these basic elements in series and/or in parallel, we can recreate an entire pathway. Each described pathway constitutes a block chain that can be connected to other block chains in case of shared metabolites. The block structure is user-friendly and highly expandable. It is easy, indeed, to define new cell types by adding or removing some blocks just as we did in previous works, when we created ENMET [17] and ADMET [18], starting from HEMETβ. Otherwise, single virtual cell models can be merged adequately to mimic their interaction *in vivo* and/or *in vitro*, as we did developing CREPE [19] focusing on the effect that endothelin-1 (secreted by endothelial cells) has on the hepatic glucokinase activity.

**Figure 1:**
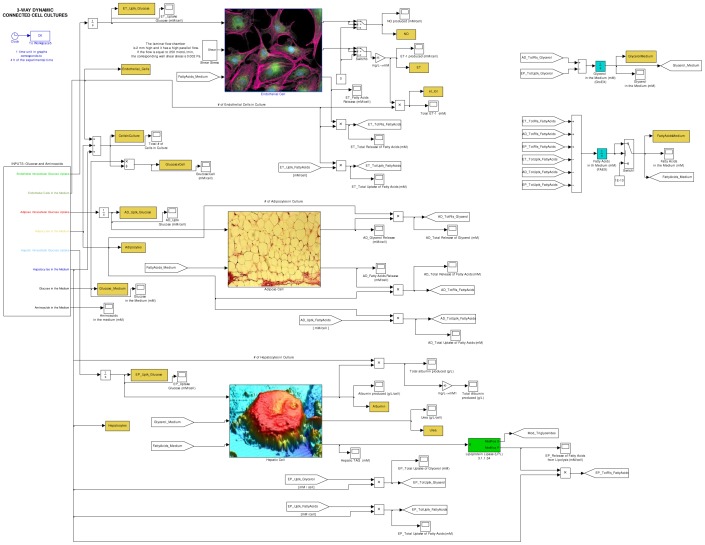
The main interface of the complete (3-way connected) model developed in this work. There are three principal blocks, represented by cell images: from top to bottom, they correspond to networks of reactions describing endothelial, adipose and hepatic metabolism. On the right side, it is possible to see blocks simulating extracellular fatty acid and glycerol concentrations.

Here is a synthesis of the main metabolic pathways already reproduced in the previous three different virtual cells. HEMETβ model describes the hepatic cell metabolism in standard conditions (cell culture in a plastic multi-well placed in an incubator at 37°C with 5% of CO_2_) and with excess substrates concentration, hence considering cell culture proliferation, nutrient uptake glycolysis, pentose phosphate pathway, degradation of proteins, urea production, glycogen, fatty acid and albumin syntheses, whereas excluding the other metabolic pathways, such as β-oxidation. ENMET mimics the same principal metabolic pathways, with the exclusion of glycogen and albumin synthesis and the addition of shear stress generation, nitric oxide production and endothelin-1 secretion, connecting mechanical stimuli responses (i.e., vasoactive substances production) to other biochemical reactions. ADMET includes the following metabolic pathways: glucose and aminoacid uptake, glycolysis, pentose phosphate pathway, Krebs cycle, aminoacid degradation, fatty acid and triglyceride synthesis, lipolysis, and the energy function. In particular, this model mimics the behaviour of a human white fat cell that responds to various compositions of the culture medium, with glycerol and free fatty acid release, which are the main indicators of fat cell activity. In the present work, we focused our attention on the integration of metabolism for the three different cell phenotypes and we dealt with these metabolic pathways: a suitable revision of cell culture proliferation models, the maintenance of glycemic balance, the uptake or release processes for specific metabolites, the triglyceride/free fatty acid cycle and glyceroneogenesis. These topics are individually discussed below. The additions regarding detailed carbohydrate metabolism and other metabolic aspects were necessary to describe interactions among different cell types. To take a couple of examples: we had to implement anabolic pathways, such as gluconeogenesis, to account for a glucose release process; similarly, we had to introduce glycerol metabolism pathway to validate our model against experimental glycerol concentration data. Novelties and improvements introduced with regard to the model presented are described in details in the following sections. **Fig. 1** shows the main interface of the complete model (for further details on the state variables and the state equations employed in our model, see **S1**, **S2** and **S3 Tables**).

### 2. Cell Proliferation Models

In HEMET, HEMETβ and ENMET cell proliferation was modelled with a logistic function, which is characterized by the following general quadratic form:
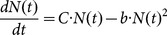
(6)where 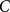 and 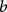 are respectively cell growth rate and death rate. In ADMET, adipose population was considered constant over time. In CREPE, a less compact equation (derived from Equation (6)) was implemented and its analytical expression is reported here below with respect to endothelial cell growth:
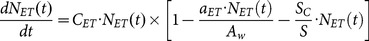
(7)In equation (7), the death term correlates both with contact inhibition (second term in square brackets) and substrate lack (third term in square brackets). For further details on the significance of each coefficient and subscript, please see ref. [21].

In this work, we defined a cell proliferation model for each cell phenotype considered. Available literature data were collected, looking for works with methodological consistency in terms of cell lines and culture conditions. These data were extracted through graphical analysis and then elaborated with Curve Fitting Toolbox (The MathWorks, Inc.) to test hypotheses and estimate parameter values.

Numerical data about hepatocytes derived from the study [31], where HepG2 immortalized cell line was used as the experimental model both in static and dynamic (i.e., with medium flowing through the bioreactor) culture conditions. Upon validating the logistic model (Equation (6)) against these data and estimating parameter values, we defined the final form of the logistic equation to implement as the one published by Verhulst [32] in 1838:
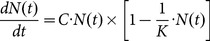
(8)where 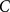 is the proliferation rate (h^−1^) and the negative term relating to 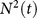 accounts for contact inhibition phenomena, which are typical of HepG2 cells. In particular, 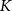 is the carrying capacity of the system. We also considered the absence of a delay term at the start of proliferation (thinking of hepatic cells as already adapted to culture conditions) and the absence of a death term due to substrate lack (supposing nutrients were sufficient for the short duration of the experiments).

Estimated mean coefficient values were used for the implementation phase: 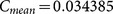 h^−1^ and 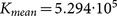 cells.

Data about endothelial cells proliferation came from the study [33], where baseline experiments were conducted comparing HepG2 and endothelial population behaviour: results showed that endothelial cell proliferation was negligible if compared with the one of hepatocytes, other things being equal. In addition, cultures started with settled cells and, because of the 48 h duration of the same experimental tests, endothelial cells did not show contact inhibition phenomena, nor underwent substrate lack [33]. Taken together, these observations led us to consider endothelial population constant over time and equal to the number of cells initially seeded 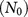.

Adipocytes were similarly treated: experiments considered in the subsequent validation phase involved only mature adipocytes, while culture conditions and the duration of experiments did not allow cells to differentiate, as well explained and confirmed by cell counting tests in the work [34]. Therefore, adipose population was considered time-invariant and equal to its initial value 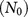.

For endothelial cells and adipocytes, cell proliferation model was described by the following equation:
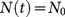
(9)Seeding values for different cell phenotypes are specified below.

These simple equations about cell proliferation may clash with the detailed enzymatic step introduced in the model (see below), but we choose not to take into account other aspects, e.g. turn over of different metabolites, in order to make our final model less complex. We instead focused our modelling efforts on metabolic pathways. Equations (8) and (9) were implemented for static as well as for dynamic cultures, according to what stated in the work [23], which we selected for the validation phase: Vinci *et al.* did not report numerical data about cell growth, but they affirmed that cell counting tests were performed demonstrating that the dynamic flux did not influence the examined cell proliferation processes.

### 3. Integrated Metabolism

The main organs taking part in energy metabolism differ in their specific enzyme content: each one is specialized for storing, using or producing different kinds of energy substrates such as triglycerides (TGs), proteins or glycogen. Because glucose and fatty acids are alternative substrates, sometimes competing, many interactions among metabolic pathways involve them [35]. This type of inter-organ cooperation is afforded by an intense and coordinated metabolic cross-talking.

For example, one of the main roles of the liver is the maintenance of glycemic levels: this organ is able to perceive a fasted state and then enhance glucose synthesis and its exportation to other tissues. In co-cultures described in [47], hepatocytes were shown to reduce their glucose uptake rate, in favour of glucose dependant cell populations. As discussed later, this homeostatic behaviour was tested in the validation phase of our workflow. Concerning lipid metabolism instead, an enzyme with a key-role is lipoprotein lipase [48], which is localized on the luminal surface of endothelium and is responsible for the hydrolysis of circulating TGs, once they are released by the hepatic tissue.

#### 3.1. Fatty Acid and Glycerol Transport Mechanisms

The transportation of molecules through the cell membrane bilayer is of paramount importance for the organism vitality [35]. Here, we focus on two kinds of transport proteins and on their role in homeostatic mechanisms.

The liver is one of the main regulators of metabolite flux, removing metabolites from blood or releasing them in it. In particular, fatty acid uptake phenomena in hepatocytes have been intensely investigated, for example [36]. Circulating free fatty acids (FFA), coming from lipolysis of stored TGs in adipocytes and from dietary fat, are an important source of lipids for the hepatocytes. Once in hepatocytes, FFA may either undergo β-oxidation in mitochondria, to produce both energy for the cell and ketone bodies, or be converted to TGs, which can be used for the production of very low-density lipoproteins, then exported. Excess TGs may be stored in lipid droplets.

Kinetic studies, employing almost-physiological conditions, have shown that FFA uptake rates can best be interpreted as the combination of a saturable component and a linear non-saturable one [37] and that the uptake rate depends on the unbound FFA concentration. However, the values for diffusion constant are generally very small if compared to those typical of facilitated transport parameters, thus the contribution of diffusion to uptake rate may be reduced or neglected in an analytical model [38]. Therefore, the total FFA uptake by hepatocytes depends on both the concentration of FFA in plasma and the capacity of the cells for FFA uptake. For hepatic and adipose tissues, various proteins mediate fatty acid transport through the membrane. One of the best known is fatty acid translocase (FAT, also referred to as CD36) [39] [40]. In physiological concentration conditions for total plasma fatty acids (bound and unbound, 0.45 mM), saturation kinetic prevails and it is well described by a Michaelis-Menten model equation:
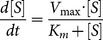
(10)where 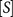 is the unbound FFA concentration and 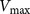 and 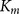 are kinetic parameters relating to protein mediated transport, in analogy to what seen for enzymes.

In this work, in order to simplify fatty acid transport modelling, only a saturable kinetic was chosen with the mediation of a single kind of protein, CD36, for all three cell phenotypes involved. Kinetic parameters for long-chain fatty acids were studied for adipocytes, hepatocytes [37] and endothelial cells [41] through [^3^H]-oleate uptake assays. As shown in **Table 1**, we used these measurements, adequately elaborated, to implement our *in silico* model.

**Table 1:** Fatty Acid Translocase kinetic parameters for hepatic (CD36_EP_), endothelial (CD36_ET_) and adipose (CD36_AD_) cells in a saturable process.

Cellular Fatty Acid Translocase	Function	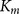 (mM)	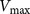 (mM h^−1^cell^−1^)	Reference
CD36_EP_	Fatty acid uptake	113·10^−6^	8.76	[37]
CD36_ET_	Fatty acid uptake	25·10^−6^	0.17	[51]
CD36_AD_	Fatty acids uptake	101·10^−6^	0.0025	[37]

In our body, adipocytes are one of the major sources of glycerol, which is in turn one of the main substrates of hepatic gluconeogenesis [42]. Briefly, during fasting, energy stored as TGs in adipocytes, is made available to other tissues by release of fatty acids, which are exported by special fatty acid transporters, and of glycerol, which is exported through suitable porins referred to as aquaglyceroporins type 7 (AQP7). The preferred hepatic gluconeogenic substrate, glycerol, directly flows in the liver via the portal vein and passes through aquaglyceroporin type 9 (AQP9) reaching the hepatocyte cytoplasm, where it is converted into glycerol-3-phosphate. This is in turn a substrate for gluconeogenesis or, along with fatty acids, it is then esterified to TGs. Multiple research groups have reached the same conclusion: these two proteins (AQP7 and AQP9) form an axis for energy transfer characterized by a coordinated regulation [42]. Glycerol transport through aquaglyceroporins pores is an example of facilitated diffusion and it is driven by the concentration gradient existing between extracellular and intracellular compartments [43]. When molecules diffusion through plasma membrane is analysed, Fick's law often takes on this form:
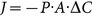
(11)where 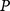 (cm/s) is the permeability coefficient of the membrane for a given substance and can be experimentally defined. This coefficient includes the diffusion coefficient 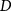 (cm^2^/s) and the membrane thickness 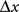 (cm), while 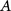 is the membrane area and 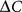 stands for the concentration difference. Glycerol permeability values for cell membranes expressing AQP7 and AQP9 can be found in literature as defined through [^14^C]-labelled solute assays and radioactivity measurements. In this work, we assumed a diffusive process thoroughly mediated by aquaglyceroporins to mimic glycerol transport in hepatic and adipose tissues. Permeability coefficients for AQP7 [44] and AQP9 [45] [46] were calculated starting from available glycerol uptake rate values and surface to volume ratios for cells, as reported in **Table 2**.

**Table 2:** Aquaglyceroporin permeability values for hepatic (AQP9) and adipose (AQP7) glycerol transport.

Aquaglyceroporins	Function	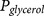 (m h^−1^)	Reference
AQP9	Glycerol uptake	0.396·10^−3^	[46]
AQP7	Glycerol release	0.09·10^−3^	[44]

#### 3.2. Glyceroneogenesis and the Triglyceride/Fatty Acid Cycle

During fasting in all mammals, triglyceride stored in adipose tissue is hydrolysed by a hormone-sensitive lipase to produce free fatty acids and glycerol. Glycerol is exported to the liver, whereas in the adipose tissue there is a considerable re-esterification of FFA depending on intracellular glycerol-3-phosphate concentration. The triglyceride/fatty acid cycle includes local intracellular recycling, within the adipose tissue, and extracellular or systemic recycling, through the formation of TGs in the liver. Intracellular recycling appears to represent almost 20–30% of the total, whereas non-adipose tissue recycling (primarily hepatic) accounts for 50% of re-esterification of fatty acids in healthy adults after an overnight fast [49]. It is clear that this cycle requires the constant generation of glycerol-3-phosphate for triglyceride synthesis.

The liver can readily use the glycerol as a source of glycerol-3-phosphate thanks to a considerable glycerol kinase activity. In adipose tissue, instead, there is a special pathway for the generation of glycerol-3-phosphate from precursors other than glucose: it is termed glyceroneogenesis and it is an abbreviated version of gluconeogenesis. Indeed, the tissue contains both pyruvate carboxylase and the cytosolic form of phosphoenolpyruvate carboxykinase. Therefore, any variation in adipocyte glucose uptake and in glyceroneogenesis flux, notably affects triglyceride/fatty acid cycle in these cells [49].

### 4. New and Specific Metabolic Pathways Introduced

#### 4.1. Hepatic Pathways

In order to reproduce hepatic cell behaviour with regard to metabolic homeostasis, we introduced the following aspects in the model: gluconeogenesis, glycogenolysis, glycerol metabolism, FFA and TG syntheses, glucose uptake and release, fatty acid uptake, triglyceride release and glycerol uptake.

Gluconeogenesis is the generation of glucose from non-carbohydrate carbon substrates such as pyruvate. In this work, only two substrates of that kind were considered: aminoacids and adipose glycerol. This way proceeds in opposite direction with respect to glycolysis and three specific enzymes counteract glycolytic irreversible kinetic steps, whereas the other seven gluconeogenic reactions are catalysed by the remaining glycolytic enzymes. The gluconeogenic enzymes introduced are pyruvate carboxylase (PYC, 6.4.1.1, irreversible), phosphoenolpyruvate carboxykinase (PEPCK-C, 4.1.1.32, reversible) and fructose-1,6-bisphosphatase (FBPase, 3.1.3.11, irreversible).

Glycogenolysis involves mobilization and degradation of glycogen stores to produce directly available energy substrates and implies the breakage of 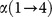 and 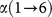 bounds. The new enzymes are glycogen phosphorilase (GPase, 2.4.1.1, reversible, *in vitro*) and α-1,6-glycosidase (A16G, 3.2.1.33, irreversible). The result is the production of glucose and glucose-6-phosphate.

As discussed above, glycerol uptake was modelled through the first Fick's law as a pore mediated (AQP9) diffusive process. Once in the hepatocyte, glycerol can become a gluconeogenic substrate through the combined action of glycerol kinase (GroK, 2.7.1.30, irreversible) and of phosphate glycerol dehydrogenase (G3PDH, 1.1.1.8, reversible). The ATP-dependent phosphorilation catalyzed by glycerol kinase gives glycerol-3-phosphate, which can also become a substrate for TG synthesis.

Fatty acid transport instead was treated with a saturation kinetic through transmembrane protein CD36. FFA and TG syntheses were then modelled following the kinetic approach reported in [18]. TGs were supposed to directly pass through the cell membrane and reach the extracellular compartment. For glucose, a bidirectional movement and a proportional term of release/uptake was implemented with regard to intracellular/extracellular glucose concentration, respectively.

**Figure 2:**
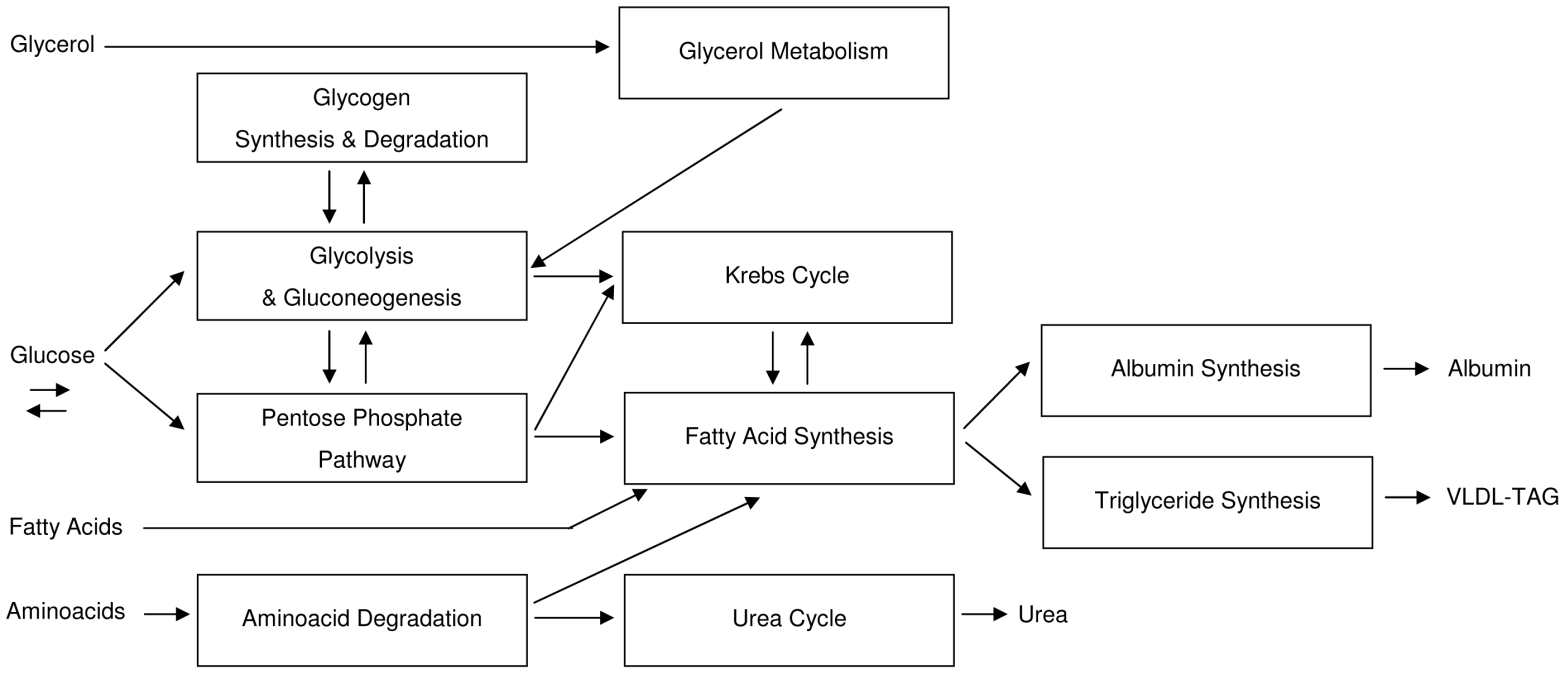
Block diagram showing an overall view of the metabolic pathways with respective interconnections implemented for hepatic cell. High-energy molecules (ATP, NADH, etc.) metabolism and energy function are not included for clarity, because they influence every subsystem.

The hepatic metabolic pathways implemented are summarized in **Fig. 2**.

#### 4.2. Endothelial Pathways

In order to reproduce endothelial cell behaviour with regard to metabolic homeostasis, we considered the following metabolic pathways: gluconeogenesis, FFA and TG syntheses, lipolytic action of lipoprotein lipase, glucose uptake and release, fatty acid uptake.

On the inner surface of capillaries, circulating TGs can be hydrolyzed to form glycerol and FFA thanks to the action of the extracellular enzyme lipoprotein lipase (LPL, 3.1.1.34, irreversible). The neighbouring cells can reabsorb energy products. This metabolic aspect is involved only in the 3-way connection scheme, where hepatic TGs act as enzymatic substrate. In regards to fatty acid release, we referred to data reported in study [51]. We supposed that only 40% of available TGs was converted into FFA and 3 nmol of the latters were obtained from each nanomole of TGs.

**Figure 3:**
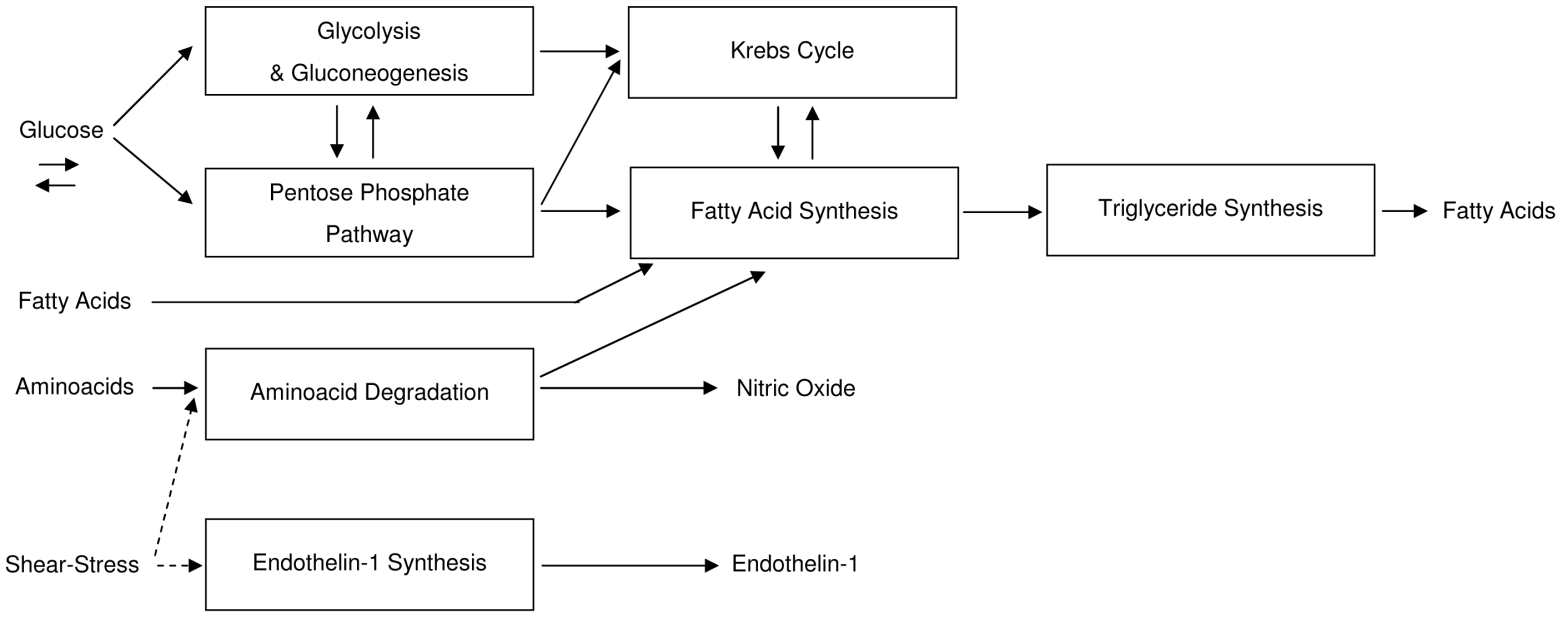
Block diagram showing an overall view of the metabolic pathways with respective interconnections implemented for endothelial cell. The model of the shear-stress acting on the cell is also reported.

All the other pathways were implemented as described above for the hepatocyte. The endothelial cell metabolism implemented is summarized in **Fig. 3**.

#### 4.3. Adipose Pathways

In order to reproduce adipocyte behaviour with regard to metabolic homeostasis, we dealt with the subsequent pathways: glyceroneogenesis, glucose uptake and release, fatty acid uptake and release, intracellular re-esterification of fatty acids, glycerol release.

For the glyceroneogenesis, we exploited enzymatic blocks already implemented in the available adipocyte model: pyruvate carboxilase, phosphoenolpyruvate carboxykinase (now reversible), and glycerol-3-phosphate dehydrogenase. The adipose intracellular component of the previously described triglyceride/fatty acid cycle accounts for about 25% of the re-esterification of the total TGs produced by the cells themselves. As done for the hepatocyte, glycerol release was modelled as a pore mediated (AQP7) diffusive process for glycerol coming from TG hydrolysis.

**Figure 4:**
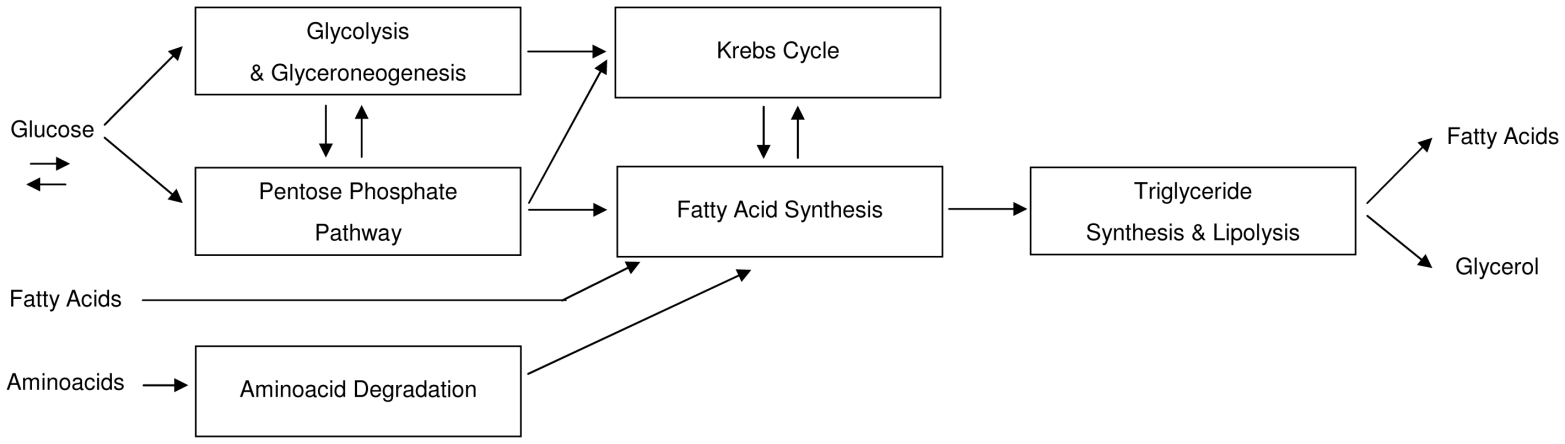
Block diagram showing an overall view of the metabolic pathways with respective interconnections implemented for adipose cell.

The whole adipocyte metabolism implemented is summarized in **Fig. 4**.

#### 4.4. Selected Simulations and Metabolite Concentrations in the Culture Medium

As we will see later, a key point of the validation phase is the comparison between simulated metabolite concentrations and the measured ones for glucose, fatty acids and glycerol. For each metabolite in the culture medium, a corresponding extracellular integrator was implemented in the model. Initial condition values were specified basing upon available experimental data [23] [24].

At first, in this work, single-cell simulations were compared with baseline monoculture experiments focusing on static conditions. Then, two kinds of configuration were studied to investigate about the metabolic homeostasis of the same *in vitro* cell systems in dynamic conditions: single-cell simulations, which were compared with baseline monoculture studies carried out in the presence of the medium flow, and simulations for a three-cell *in silico* model, which were compared with results from the 3-way connected culture system, were examined.

As the configuration changed, from single-cell model to three-cell model, so did state equations for metabolites of interest (i.e., cell specific terms were added or deleted from the equations). Extracellular metabolite concentrations for the 3-way connected system were described through the following three state equations:
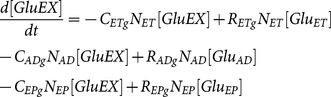
(12)




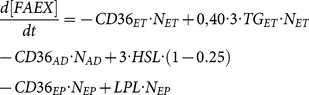
(13)




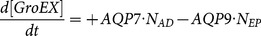
(14)where 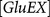, 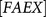 and 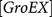 are extracellular glucose, fatty acid and glycerol concentrations respectively, 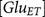, 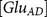 and 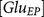 are instead intracellular glucose concentrations, 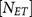, 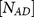
and 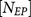 are the number of cells for the three phenotypes considered, 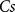 and 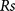 are cellular glucose uptake and release rates, respectively; the other terms are enzymatic rates for corresponding enzymes or transport proteins. Equations (12), (13) and (14) inevitably consisted of many terms: in order to reproduce metabolic interactions among different cell types, we had to consider uptake and release phenomena for mutually inter-changed metabolites. Those equations allowed us to correlate measured extracellular metabolite concentrations with metabolic processes occurring inside the cells. The same equations were exactly used for all of our simulations concerning the 3-way connected system. For the sake of simplicity, equations are reported in the text only for the complete model. Simpler equations were implemented for single-cell simulations (for details, see **S2 Table**). All the values used for new kinetic enzyme parameters are listed in **Table 3** and **Table 4**.

**Table 3:** Enzymatic parameters (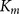 for direct 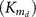 and indirect 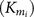
reactions, 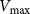, kinetic rate expressions) relating to the new enzymes introduced in the model for carbohydrate metabolism.

Enzyme	EC number	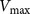 (mM h^−1^)	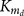 (mM)	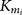 (mM)	Kinetic rate
Pyruvate carboxylase	6.4.1.1	263160	0.24 (pyruvate/ATP 1)		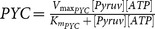
Phosphoenol-pyruvate carboxykinase	4.1.1.32	Direct: 68.058 Indirect: 57.114	0.026 (oxalacetate)	0.048 (PEP)	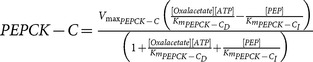 .
Fructose-1,6-bisphosphatase	3.1.3.11	139.092	0.0016 (FBP)		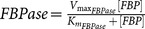
Glucose-6-phosphatase	3.1.3.9	4800·10^-9^	4.8 (G6P)	4 (Glucose) (supposed value)	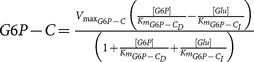
Glycogen phosphorylase	2.4.1.1	2016	1.4 (Glycogen)	1.3 (G1P)	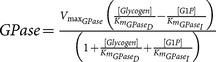
Alpha-1,6-glucosidase	3.2.1.33	660	0.063 (Glycogen)		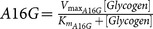

1 Abbreviations: ATP (Adenosine TriPhosphate); Pyruv (Pyruvate); PEP (PhosphoEnolPyruvate); FBP (Fructose-BisPhosphate); G6P (Glucose-6-Phosphate); Glu (Glucose); G1P (Glucose-1-Phosphate).

**Table 4:** Enzymatic parameters (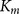 for direct 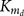
and indirect 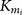 reactions, 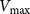, kinetic rate expressions) relating to the new enzymes introduced in the model for lipid metabolism.

Enzyme	EC number	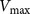 (mM h^−1^)	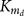 (mM)	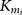 (mM)	Kinetic rate
Acetyl-CoA carboxylase	6.4.1.2	3723	0.05 (Acetyl-CoA 1)		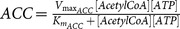
Fatty-acid synthase	2.3.1.85	19212·10^-6^	0.019 (Malonyl-CoA)		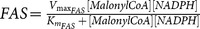
Glycerol kinase	2.7.1.30	762·10^−3^	0.046 (Glycerol)		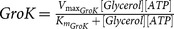
Glycerol-3-phosphate dehydrogenase	1.1.1.8	357000	0.15 (DHAP) 0.01 (NADH)	0.19 (Glycerol-3P)	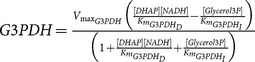
Acyl-CoA Synthase	6.2.1.3	107.175·10^−3^	0.00278 (Palmytate)		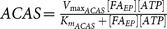
Glycerol-3-phosphate acyltransferase	2.3.1.15	3.876	0.46 (Glycerol-3P) 0.0031 (Palmitoyl-CoA)		
1-acylglycerol-3-phosphate acyltransferase	2.3.1.51	10.98·10^−6^	0.01630 (Acyl-CoA)		
Diacylglycerol acyltransferase	2.3.1.20	6723·10^−3^	0.008 (Oleoyl-CoA)		
STG (Synthesis of Triglycerides)	(cumulative)				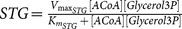
Lipoprotein lipase	3.1.1.34	2.261	0.053 (VLDL)		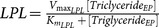

1 Abbreviations: ATP (Adenosine TriPhosphate); DHAP (dihydroxyacetone phosphate); NADH (Nicotinamide Adenine Dinucleotide Hydrogen); Glycerol-3P (Glycerol-3-Phosphate); FA_EP_ (hEPpatic Fatty Acids); VLDL (Very Low-Density Lipoprotein); Triglyceride_EP_ (hEPpatic Triglyceride).

### 5. Validation Procedure

The validation phase was based upon three studies [21] [23] [24], characterized by a logical and temporal order. The same group of authors conceived them to analyse metabolic homeostasis in the human visceral region.

The duration of all their experiments was equal to 48 h, so we assumed that one simulation time unit was equivalent to 4 h and each simulation lasted 12 time units. We used ode 23 s (stiff/Mod.Rosenbrock) as numerical integration method. The cell simulators did not take into account any change of the culture medium.

Extracellular concentration data were available from the three works for four different sampling times (0, 15, 24 and 48 h) and for glucose, fatty acid and glycerol concentrations. We extracted their numerical values exploiting tables or graphical analysis of plots reported. Extracted values were expressed as means with respective standard deviation and they were often affected by an unmodifiable large deviation [23] [24], as we will see below. In order to analyse the differences between the dynamic and static monocultures, the authors of study [23] chose to focus on the net change of metabolite concentrations in the culture medium between 0 and 48 h, as after this time cells were thought to be adapted to seeding and culture conditions. We adopted the same approach for the comparison between simulation and experimental data, focusing on the first and the last data points. As for glucose and aminoacid concentrations (input variable), we decided to use the known Eagle's MEM formulation values, as reported in **Table 5**.

**Table 5:** Data about glucose and aminoacid concentration values employed in the presented *in silico* model. They refer to Eagle's MEM culture medium formulation.

Chemical component	Concentration [mM]
L-Alanine	0.281
L-Arginine	0.723
L-Asparagine	0.605
L-Aspartate	0.990
L-Cysteine	0.620
L-Glutamate	0.510
L-Glutamine	0.007
L-Glycine	0.666
L-Histidine	0.271
L-Isoleucine	0.396
L-Leucine	0.396
L-Lysine	0.496
L-Methionine	0.101
L-Phenilalanine	0.194
L-Proline	0.347
L-Serine	0.238
L-Threonine	0.403
L-Tryptophan	0.049
L-Tyrosine	0.286
L-Valine	0.393
D-Glucose	5.551

Currently, as for intracellular metabolite concentration, quantitation of all the metabolites in a cellular system in a given state at a given point in time is impossible, because of the lack of simple automated analytical strategies that can effect this in a reproducible and robust way. The main challenges are the chemical complexity and heterogeneity of metabolites, the dynamic range of the measuring technique, the throughput of the measurements, and the extraction protocols [50]. Using mass spectrometry, comprehensive surveys of cell metabolite concentrations have been made, but they are available only for *Escherichia coli* and *Saccharomyces cerevisiae*. In our model, intracellular metabolite concentrations were modelled through corresponding integrator blocks: given the lack of precise data, we hypothesised that cells were empty at the beginning of the simulations and we therefore set the initial condition value to zero for each intracellular integrator.

## Results and Discussion

### 1. Identification of Correction Factors and Validation for Cell Monocultures

First of all, we validated single-cell models against data from baseline studies [23], in which the effect of the medium flow on metabolic behaviour was evaluated, thus distinguishing static and dynamic conditions. During baseline experiments [23], 250•10^3^ hepatocytes and 200 mg of adipose tissue (about 300•10^3^ cells) were cultivated in static wells or inserted in modular bioreactor chambers (MCmB 2.0) with extremely low shear stress for cell cultures. 80•10^3^ endothelial cells were seeded in static conditions or transferred to a laminar flow chamber for dynamic tests.

**Figure 5:**
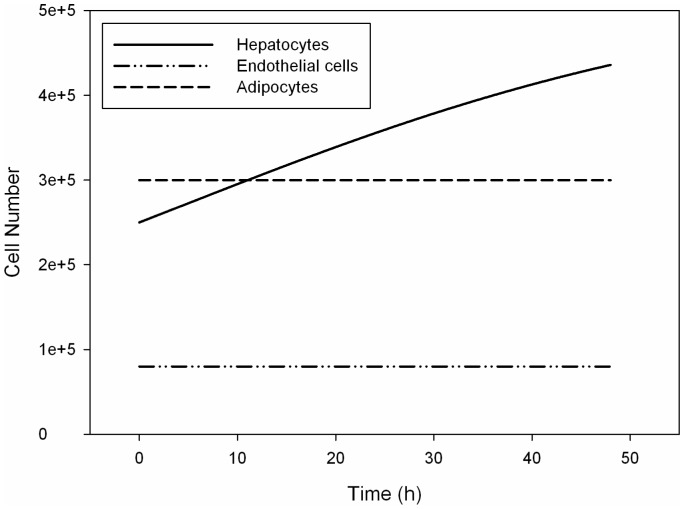
The simulated cell growth profiles in function of time. These profiles result from the implementation of the proliferation models discussed in the text. Solid line, dash-dotted line and dashed line represent hepatocyte number, endothelial cell number and adipocyte number, respectively.

Proliferation data were not available from the same work for a comparison with simulated data. We implemented the proliferation models discussed above. Then, according to what is affirmed in [23], where flow is said not to influence mitogenesis, we assumed cell growth simulation results to be valid for both the conditions examined. **Fig. 5** shows different cell growth profiles, which characterize our *in silico* models.

As far as metabolic profiles are concerned, a new pop-up variable to select was introduced *in silico* aiming to reproduce the difference between the presence (dynamic conditions) and absence (static conditions) of the culture medium flow (**S1(A) and S1(B) Figures**). For each case tested, a different set of initialization values was associated to that variable: the sets consisted of heuristically estimated correction factors that were applied to enzyme kinetic parameters (**S1(C) Figure**). The function of the correction factors was to modify selectively kinetic parameters according to different culture conditions and to reproduce the main features of metabolic profiles experimentally observed. The identification of correction factors to apply allowed us to make hypotheses about the differential activation of metabolic pathways in the culture conditions considered.

#### 1.1. Hepatocyte Monoculture

**Figure 6:**
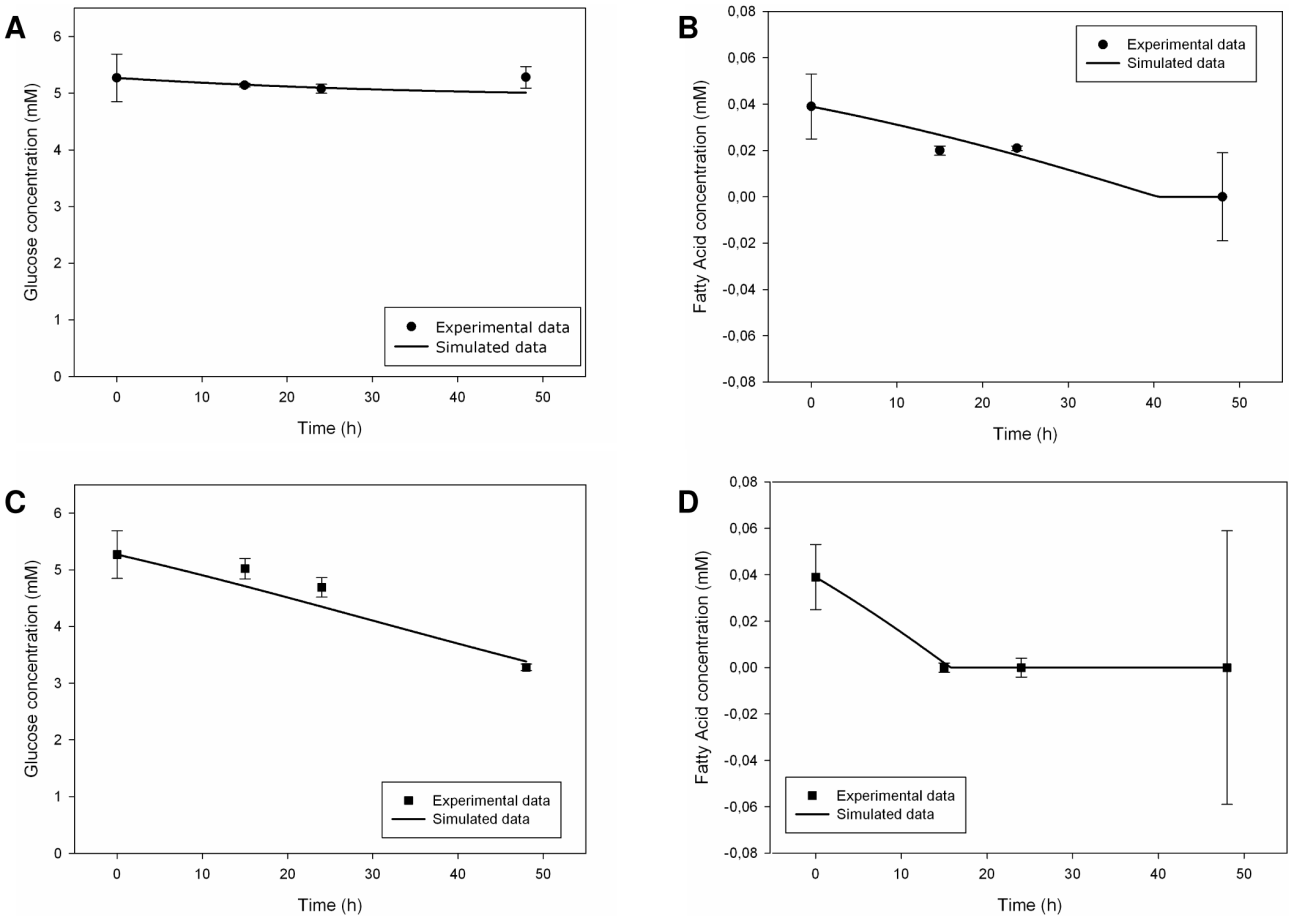
Measured [23] and simulated glucose and fatty acid trends in the culture medium for hepatic monocultures. Upper figures refer to static conditions, the other ones describe dynamic conditions. Solid lines represent the simulated data, while circles (for the static case) and squares (for the dynamic case) represent the corresponding experimental data. Measured values are expressed as means ± standard deviation for experiments run at least in triplicate: numerical values are reported in [23] and error bars represent the standard deviation. (A) Glucose trend in static conditions. (B) Fatty acid trend in static conditions. (C) Glucose trend in dynamic conditions. (D) Fatty acid trend in dynamic conditions.

For hepatocyte monocultures, we obtained a general and good agreement between experimental and simulated behaviours in the static as well in the dynamic situation and **Fig. 6(A), 6(B), 6(C) and 6(D)** show that. With regard to glucose concentration, no net change was experimentally observed in static conditions, but there was a significant cellular glucose uptake in the presence of flow [23]: we modelled this difference through an over-regulation of glucose uptake rate in the dynamic case. During the experiments, fatty acid uptake was present in both conditions with a complete removal over time, which was more rapid in the dynamic setup [23]. *In silico*, we obtained the same behaviour through an adequate regulation of kinetic parameters for both glucose and fatty acid uptake processes. *In vitro*, hepatocytes showed glycerol uptake over time, principally in dynamic conditions [23]: as suggested by the authors of the experimental study, aquaglyceroporins probably play a key-role in this uptake process. We indeed concentrated on the implementation of AQP9 and the regulation of its kinetic parameters, so obtaining fair results (**S2(A) and S2(B) Figures**).

#### 1.2. Endothelial Cell Monoculture

**Figure 7:**
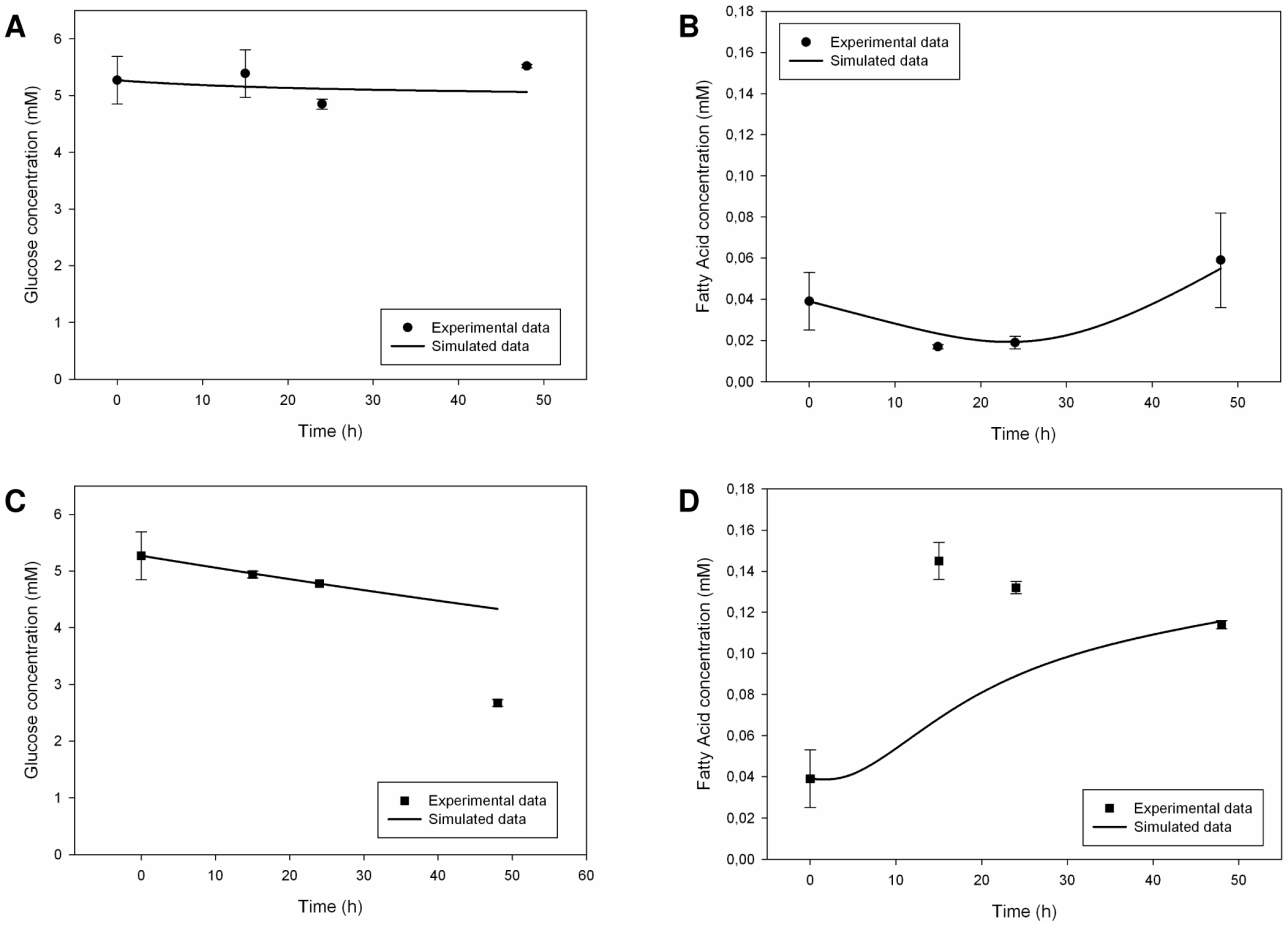
Measured [23] and simulated glucose and fatty acid trends in the culture medium for endothelial monocultures. Upper figures refer to static conditions, the other ones describe dynamic conditions. Solid lines represent the simulated data, while circles (for the static case) and squares (for the dynamic case) represent the corresponding experimental data. Measured values are expressed as means ± standard deviation for experiments run at least in triplicate: numerical values are reported in [23] and error bars represent the standard deviation. (A) Glucose trend in static conditions. (B) Fatty acid trend in static conditions. (C) Glucose trend in dynamic conditions. (D) Fatty acid trend in dynamic conditions.

The endothelial cell simulator included shear stress parameters in dynamic conditions. As far as glucose and fatty acid metabolism was concerned, the model was able to reproduce the mean experimental behaviour observed [23], only in static conditions, as shown in **Fig. 7(A) and 7(B)**. It was more difficult to mimic glucose uptake and fatty acid release in dynamic conditions, probably because of our hypothesis that cell proliferation was absent. There was only a reproduction of the general trend of the behaviour described in [23] (**Fig. 7(C) and 7(D)**). Fatty acid synthesis, uptake and consumption were indeed implemented, but we had to define the intensity of these processes under precautionary assumptions in consequence of the uncertainty of the available literature data [51]. Besides this, the zero initial condition for intracellular fatty acid integrator did not allow the cell to release a metabolite without having it right inside before. Glycerol concentration profiles were neglected because in study [23] they were not considered significant for the energy metabolism of endothelial cell, but only for the cell permeability status.

#### 1.3. Adipocyte Monoculture

**Figure 8:**
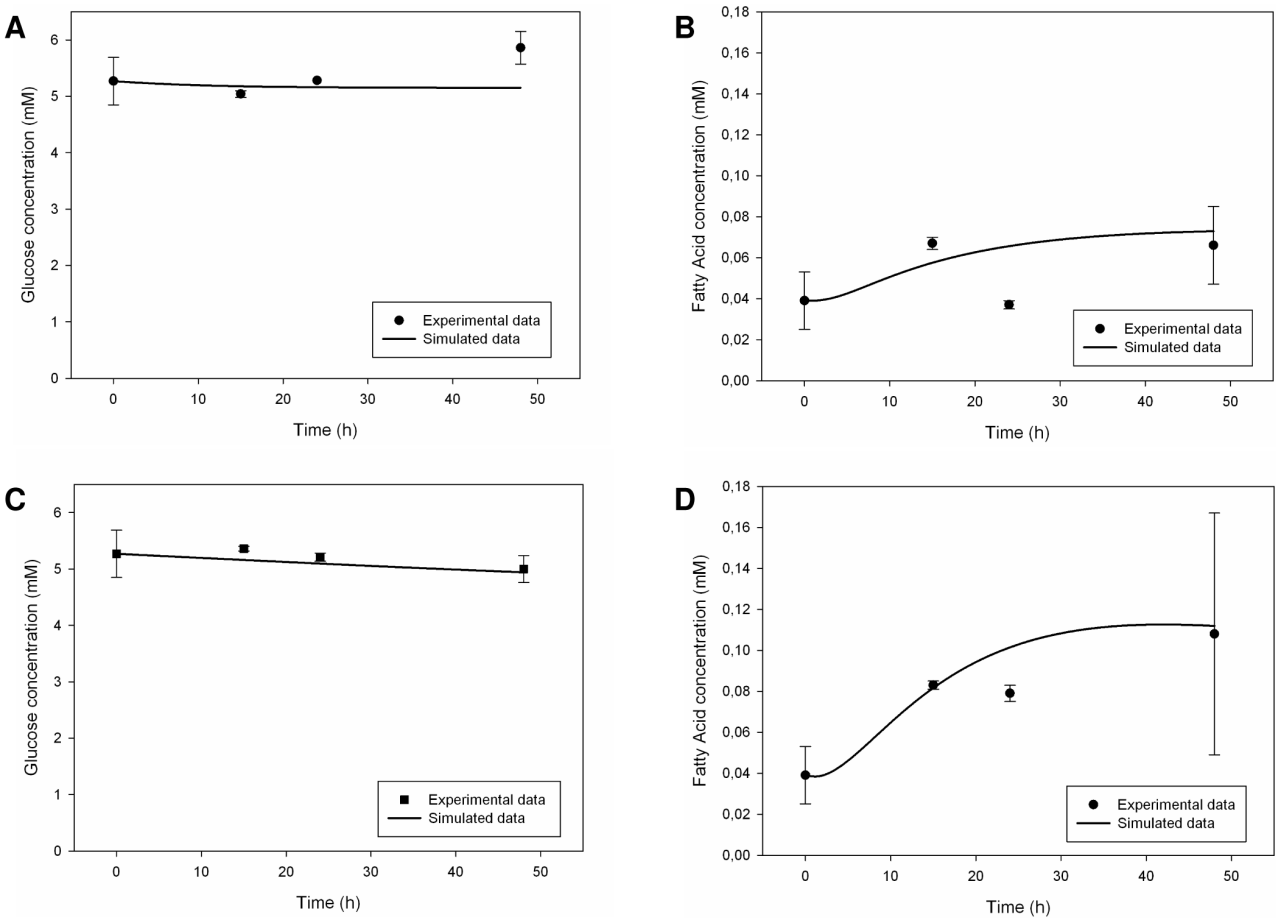
Measured [23] and simulated glucose and fatty acid trends in the culture medium for adipose monocultures. Upper figures refer to static conditions, the other ones describe dynamic conditions. Solid lines represent the simulated data, while circles (for the static case) and squares (for the dynamic case) represent the corresponding experimental data. Measured values are expressed as means ± standard deviation for experiments run at least in triplicate: numerical values are reported in [23] and error bars represent the standard deviation. (A) Glucose trend in static conditions. (B) Fatty acid trend in static conditions. (C) Glucose trend in dynamic conditions. (D) Fatty acid trend in dynamic conditions.

For adipocytes, the simulation results had a fair agreement with experimental data, principally in dynamic conditions. In the adipocyte culture, glucose concentration was stable in static conditions whereas there was a net glucose uptake in the dynamic ones, probably due to the effect of the culture medium flow [23]. *In silico*, we indeed regulated glucose metabolism parameters. Fatty acid release was present in both static and dynamic experimental conditions [23] and our model was able to reproduce it, through a distributed regulation of key-enzymes for glucose and lipid metabolism (**Fig. 8(A), 8(B), 8(C) and 8(D)**). Glycerol was also released in the culture medium both in static and dynamic conditions, as a probable consequence of constitutive lipolysis phenomena [23]. Our simulator was able to mimic the general trend, but not the intensity of the metabolite release (**S3(A) and S3(B) Figures**). Once again, the implementation of aquaglyceroporin-mediated glycerol transport improved the results. It was not possible to enhance glycerol release further due to the general consistency of the model. As already seen for endothelial cells, zero initial condition for intracellular glycerol integrator was a drawback for the model itself.

### 2. Validation for the 3-Way Connected System: Not just a Sum

The *in silico* model was subsequently validated involving the examined three cell phenotypes at the same time. We referred to experimental data reported in [21] and [24] for a 3-way connected *in vitro* culture system aiming to reproduce the metabolic homeostasis of the visceral region. This system involved hepatocytes, endothelial cells and adipocytes: 250·10^3^ hepatocytes and 50·10^3^ adipocytes were inserted in modular bioreactor chambers (MCmB 2.0) with extremely low shear stress for cell cultures, whereas 25·10^3^ endothelial cells were transferred to a laminar flow chamber for dynamic tests. The chambers were connected together through the culture medium flow.

At first, in the three-cell *in silico* model, we set the values of enzymatic parameters at the same values used for monoculture simulations in dynamic conditions, even if a small reduction of fatty acid metabolism for adipocytes was necessary in order to maintain the consistency of the model.

Experimental data showed the presence of a homeostatic regulation mechanism: changes in glucose concentration were negligible, with hepatic tissue preserving normal glucose levels [24]. Probably, hepatic gluconeogenic production of the metabolite compensated for endothelial and adipose glucose uptake: intercellular cross-talking was fundamental for this kind of metabolic control to realize. A similar experimental homeostatic balance was observed for medium fatty acid concentration with negligible changes over time [24]: the presence of the hepatic cell line prevented it from rising, probably through the removing action seen in monoculture test. Experimental glycerol concentration did not show significant variations, consistently with fatty acid trend. The presence of hepatocytes maintained glycerol balance and the authors [24] assumed a mutual metabolite inter-change among cell types as the possible explanation.

**Figure 9:**
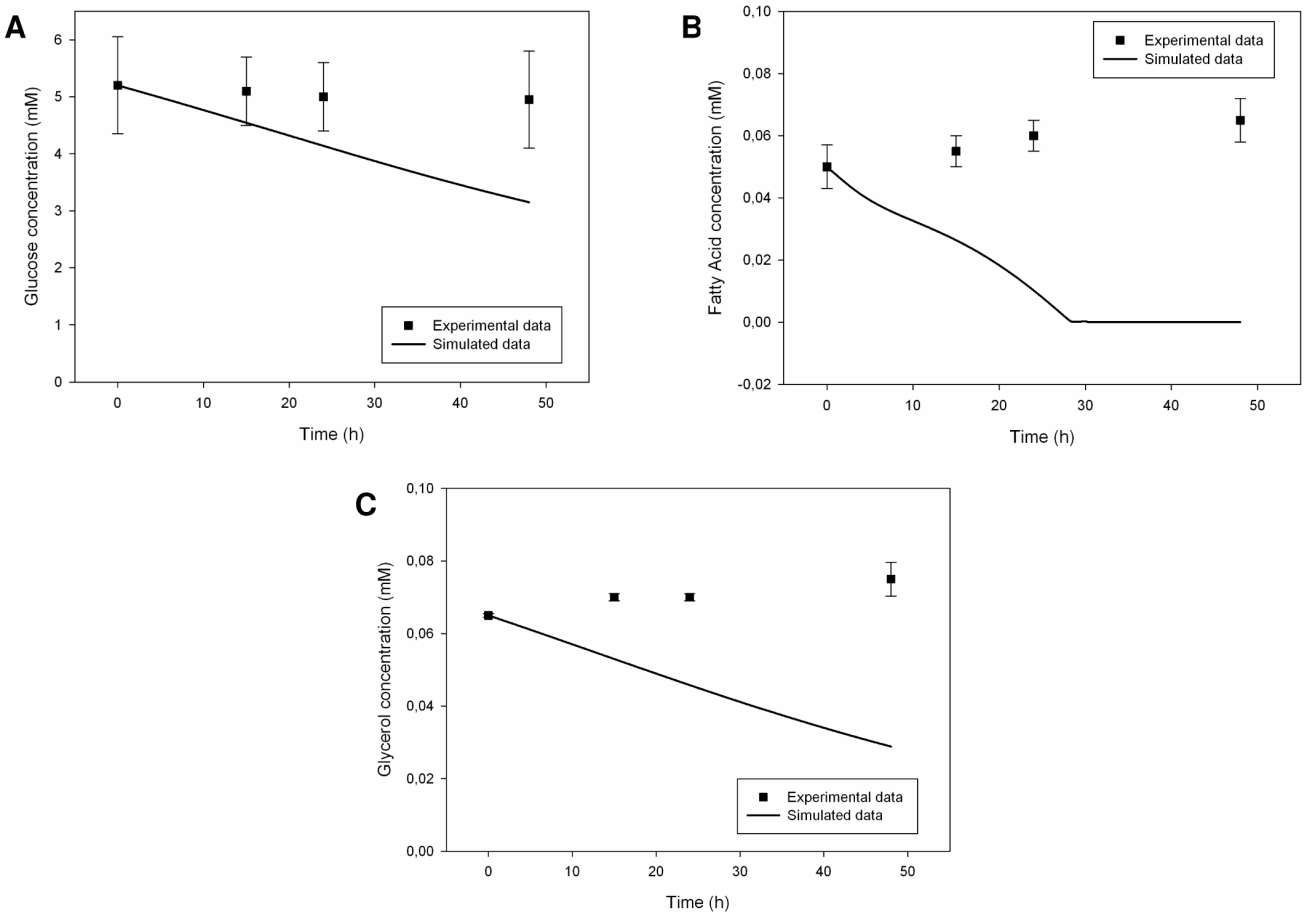
Measured [24] and simulated metabolite trends in the culture medium for the 3-way connected system (dynamic conditions). Solid lines represent the simulated data, while squares represent the corresponding experimental data. Measured values are expressed as means ± standard deviation: numerical values were extracted from plots reported in [24] and error bars represent the standard deviation. From three to six replicates were run for each experiment. (A) Glucose trend in culture medium. (B) Fatty acid trend in culture medium. (C) Glycerol trend in culture medium.

Corresponding simulation results differed from experimental observations, showing only a cumulative glucose uptake with respect to dynamic monocultures, a complete removal of free fatty acids in the medium in contrast to a small release from endothelial and adipose cultures, and a net glycerol decrease in the medium as it is typical of the hepatic population. It was evident that the hepatic population played a leading role in the shaping of the overall metabolic profile. Moreover, the zero-initial conditions for intracellular integrators had a large effect on it (**Fig. 9(A), 9(B) and (C)**).

Ultimately, the *in silico* model for the 3-way connected system was not able to reproduce the metabolic behaviour and homeostatic regulation observed *in vitro*, neither for the trends nor for the degree of variations in metabolite temporal profiles. This was explained looking at enzymatic parameters. *In vitro*, there was an evident adaptation of the metabolic balance to fluid dynamic conditions and nutrient availability, through a differentiated activation of specific metabolic pathways. The same mechanisms could not take place *in silico*: the values of the kinetic parameters were fixed at the beginning of simulations and metabolite concentrations alone were not sufficient to direct homeostatic regulation. Running different simulations has highlighted the key-role of gluconeogenesis and glyceroneogenesis enzymatic parameters with regard to fatty acid biosynthesis. This is in agreement with the physiological “de novo” lipogenesis, which is the synthesis of fatty acid molecules from non-lipid substrates, mainly carbohydrates.

### 3. Modifications for the Simulated 3-way Connected System

**Figure 10:**
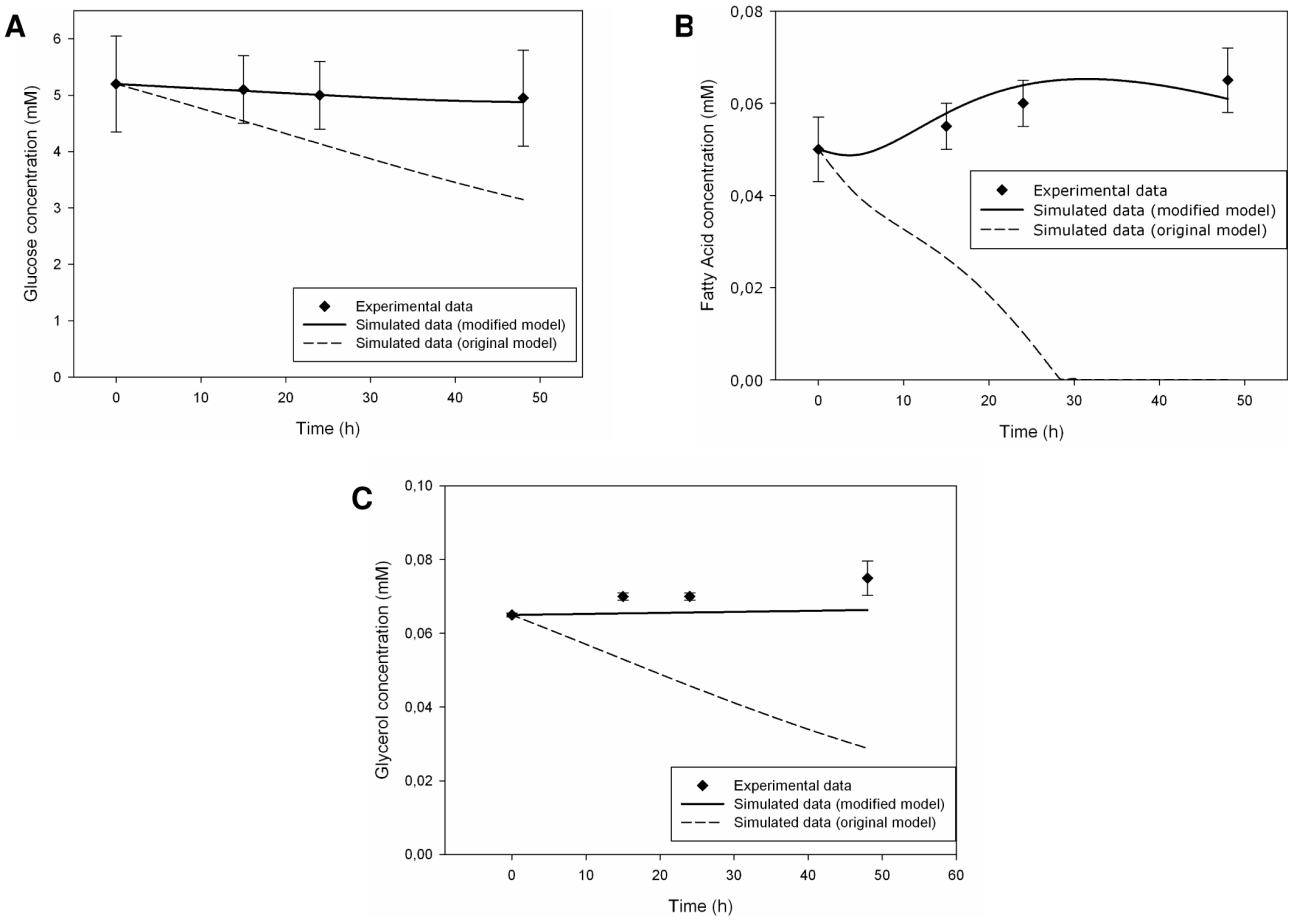
Measured [24] and simulated metabolite trends in the culture medium for the in-silico modified 3-way connected system. Parameter modifications described in the text were applied to the initial 3-way connected system model. Solid lines represent the simulated data for the modified model, dotted lines refer to the simulated data for the original *in silico* model (see **Figure 9** for details), while squares represent the corresponding experimental data. Measured values are expressed as means ± standard deviation: numerical values were extracted from plots reported in [24] and error bars represent the standard deviation. From three to six replicates were run for each experiment. (A) Glucose trend in culture medium. (B) Fatty acid trend in culture medium. (C) Glycerol trend in culture medium.

The simulation results described above would seem to indicate and reaffirm that the experimental 3-way connected system is not just a sum of the three distinct monocultures involved, but it is a complex system with “emergent properties” whose origin has to be identified. Equations (12), (13) and (14) seemed not to be sufficient for the intended modelling if the same enzymatic parameters, used for the single-cell simulators, were employed. Thus, we tried to modify some key-parameter seeking for a more effective validation of the *in silico* model. An attempt was made, basing on the biochemical and model knowledges acquired. We thought that hepatic predominance was too strong in the 3-way connected *in silico* model and we tried to reduce its contribution, modifying only few parameters. We lowered hepatic glucose, fatty acid and glycerol uptake rates to enhance nutrient availability for the other cell types. Referring to [47] we assumed that, in normoglycemic conditions, hepatic tissue could lower its glucose uptake rate to pander to cell populations that preferred glucose as energy substrate. A comparison of the parameters values employed in the two different models of the simulated 3-way connected system is reported in **Table 6**. The implementation of the modified model produced better results, as shown in **Fig. 10(A), 10(B) and 10(C)**. Simulated data agreed well with experimental ones, especially for glucose and fatty acid concentrations. Glycerol profile was affected by the scarce release from endothelial and adipose cells.

**Table 6:** Parameter values employed in the two different models of the simulated 3-way connected culture system described in the text.

Description of the parameter	Simulated 3-way connected system deriving from monoculture models	Modified simulated 3-way connected system
Hepatic glucose uptake rate	4.784·10^−8^ h^−1^ cell^-1^	4.784·10^−10^ h^−1^ cell^−1^
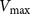 for hepatic fatty acid transporter CD36	8.755 mM h^−1^ cell^−1^	1.751 mM h^−1^ cell^−1^
Hepatic membrane permeability to glycerol	3.960·10^−4^ m h^−1^ cell^−1^	3.960·10^−7^ m h^−1^ cell^−1^
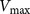 for adipose fatty acid transporter CD36	2.5·10^−3^ mM h^−1^ cell^−1^	2.5·10^−4^ mM h^−1^ cell^−1^

We believe that these results show that, with few variations, the implemented model is able to reproduce the metabolic homeostasis characterizing the human visceral compartment. In general, results were better in the case of glucose and lipid metabolism than in that of glycerol one. Future developments of the model may be able to investigate the significance of these modifications with more accuracy.

## Conclusions

Many key organs exploit complex molecular signalling pathways and interact each other to maintain the systemic energy balance of a living organism. This balance is usually deranged in obese and diabetic patients or in the metabolic syndrome disease. Clearly, the availability of tools to better understand the metabolic cross-talking phenomena is essential for finding the most appropriate interventions to treat or prevent meatabolic diseases. We have presented a new computational modular model, which is able to reproduce the metabolic behaviour observed for connected culture systems in dynamic conditions. We have studied integrated metabolism and tried to explain possible regulation mechanisms and emergent properties coming from the combination of distinct cell types. We concentrated on the human visceral region, homeostatic nutrient balance and cross-talking phenomena involving hepatocytes, endothelial cells and adipocytes. We validated our model against experimental data concerning glucose, fatty acid and glycerol trends. To the best of our knowledge, the *in silico* model presented here is the first one to consider the effect of the flow on the co-existence of multiple cell types. The metabolic network implemented may be extended, comprising other organs with a key role for energy metabolism, such as pancreas, or adding new aspects, such as lactate metabolism or hormonal regulation. A regulation model for genetic expression should be introduced to make the system more adaptable to exogenous and endogenous stimuli, therefore overcoming the fixity of kinetic parameter values during simulations. Hybrid approaches may be attempted, integrating optimization algorithms for parameter estimation and energy constraints for cells.

## Supporting Information

S1 Figure **The graphical interface allowing the user to introduce or not the presence of the culture medium flow in the simulated model.** The mask of subsystem “Input Data” through which the user can set the binary value (0 or 1) of the pop-up variable created to distinguish the static (A) from the dynamic (B) culture conditions for cell monocultures. It is followed by a screenshot (C) of a Matlab file (.m) showing the association of the pop-up variable value to a set of initialization values for enzymatic parameters.
(TIF)

S2 Figure **Measured** [23] **and simulated glycerol trends in the culture medium for hepatic monocultures.** Upper figure refers to static conditions, the other one describes dynamic conditions. Solid line represents the simulated data, while circles (for the static case) and squares (for the dynamic case) represent the corresponding experimental data. Measured values are expressed as means ± standard deviation for experiments run at least in triplicate: numerical values are reported in [23] and error bars represent the standard deviation. (A) Glycerol trend in static conditions. (B) Glycerol trend in dynamic conditions.
(TIF)

S3 Figure **Measured** [23] **and simulated glycerol trends in the culture medium for adipose monocultures.** Upper figure refers to static conditions, the other one describes dynamic conditions. Solid line represents the simulated data, while circles (for the static case) and squares (for the dynamic case) represent the corresponding experimental data. Measured values are expressed as means ± standard deviation for experiments run at least in triplicate: numerical values are reported in [23] and error bars represent the standard deviation. (A) Glycerol trend in static conditions. (B) Glycerol trend in dynamic conditions.
(TIF)

S1 Table
**The full list of all enzymes and consumption terms used in the modelling with their corresponding equations.** In reaction kinetics (both in equilibrium and non-equlibrium conditions), oxidated-form cofactors (NAD+, FAD, ADP, NADP+) are considered in saturation, then they not play an active role on the regulation of catalysis rates. It is a good approximation, in fact they are in large quantity in cells. Enzymatic parameters: 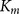 for direct 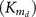 and indirect 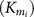 reactions, 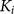 for inhibitors, 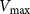 for direct 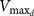 and indirect 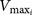 reactions.(DOCX)

S2 Table **The full list of state equations used in the modelling.**
(DOCX)

S3 Table **The full list of stoichiometric equations used in the modelling for aminoacid degradation.**
(DOCX)
