# Supplementary figures and images for: In Silico Models for Dynamic Connected Cell Cultures Mimicking Hepatocyte-Endothelial Cell-Adipocyte Interaction Circle
Source: PLoS One. 2014 Dec 15;9(12):e111946. doi: 10.1371/journal.pone.0111946 (PMC4266517; doi:10.1371/journal.pone.0111946)

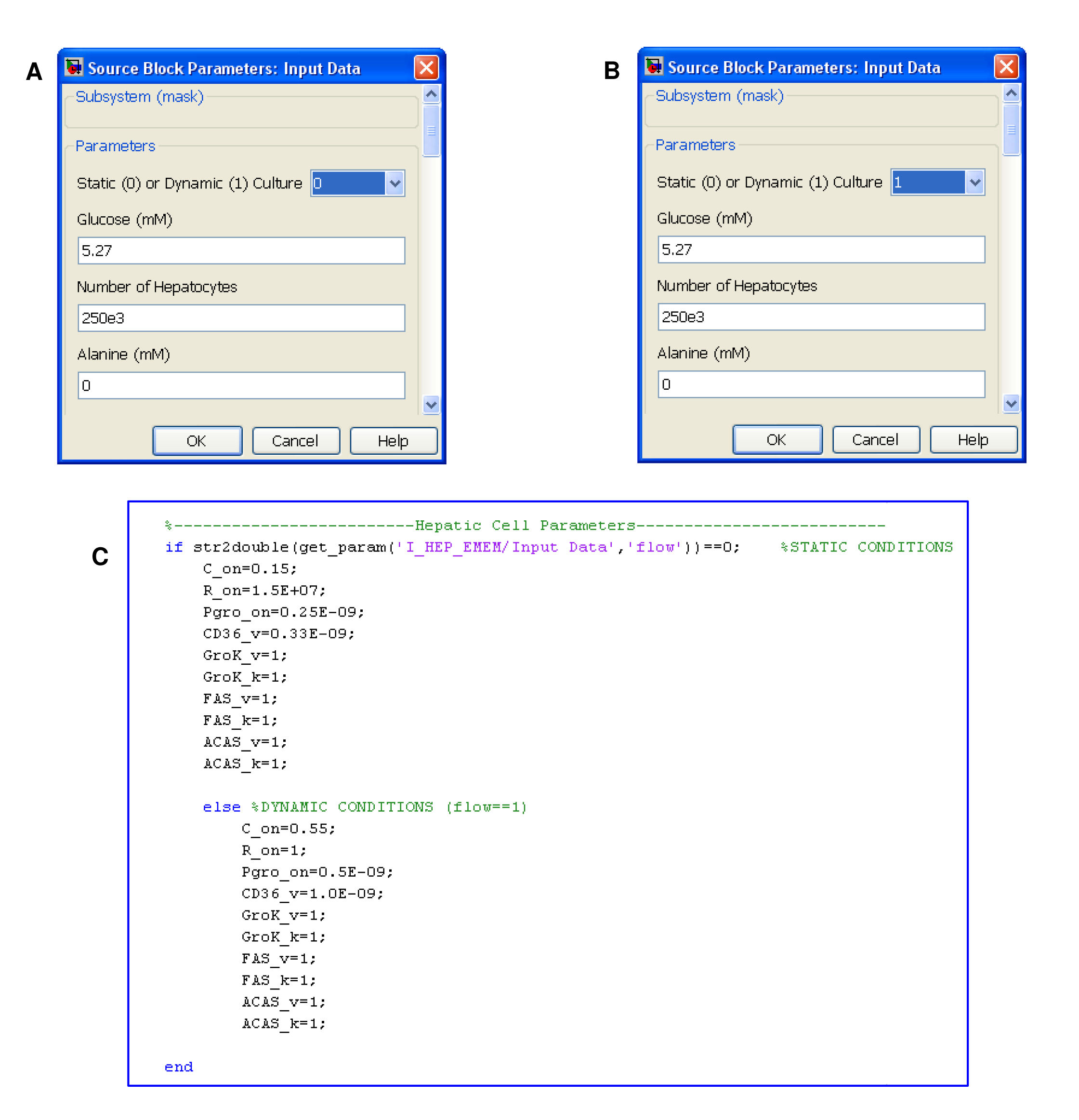

Supplement: S1 Figure — The graphical interface allowing the user to introduce or not the presence of the culture medium flow in the simulated model. The mask of subsystem “Input Data” through which the user can set the binary value (0 or 1) of the pop-up variable created to distinguish the static (A) from the dynamic (B) culture conditions for cell monocultures. It is followed by a screenshot (C) of a Matlab file (.m) showing the association of the pop-up variable value to a set of initialization values for enzymatic parameters. (TIF) [file pone.0111946.s001.tif]

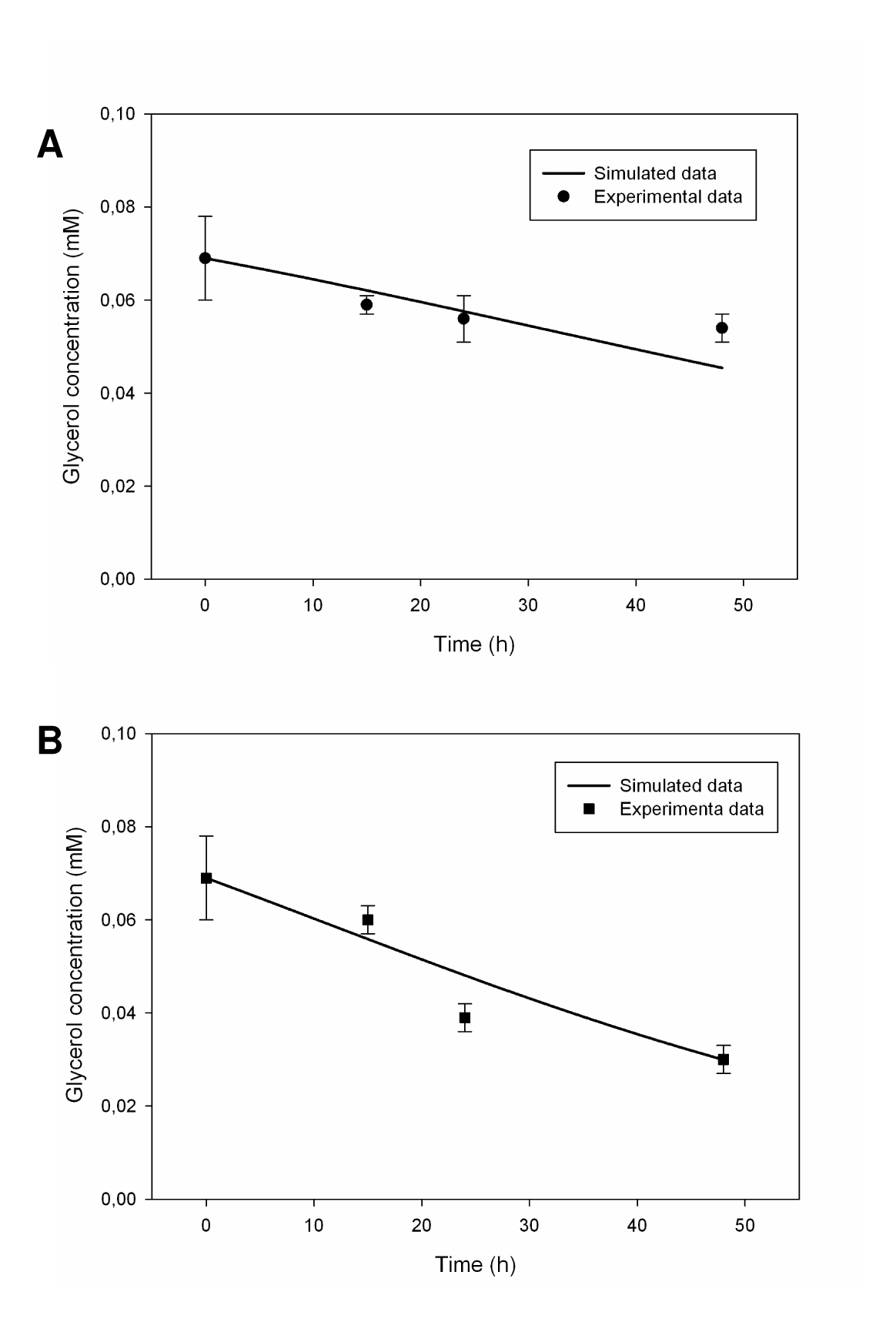

Supplement: S2 Figure — Measured [23] and simulated glycerol trends in the culture medium for hepatic monocultures. Upper figure refers to static conditions, the other one describes dynamic conditions. Solid line represents the simulated data, while circles (for the static case) and squares (for the dynamic case) represent the corresponding experimental data. Measured values are expressed as means ± standard deviation for experiments run at least in triplicate: numerical values are reported in [23] and error bars represent the standard deviation. (A) Glycerol trend in static conditions. (B) Glycerol trend in dynamic conditions. (TIF) [file pone.0111946.s002.tif]

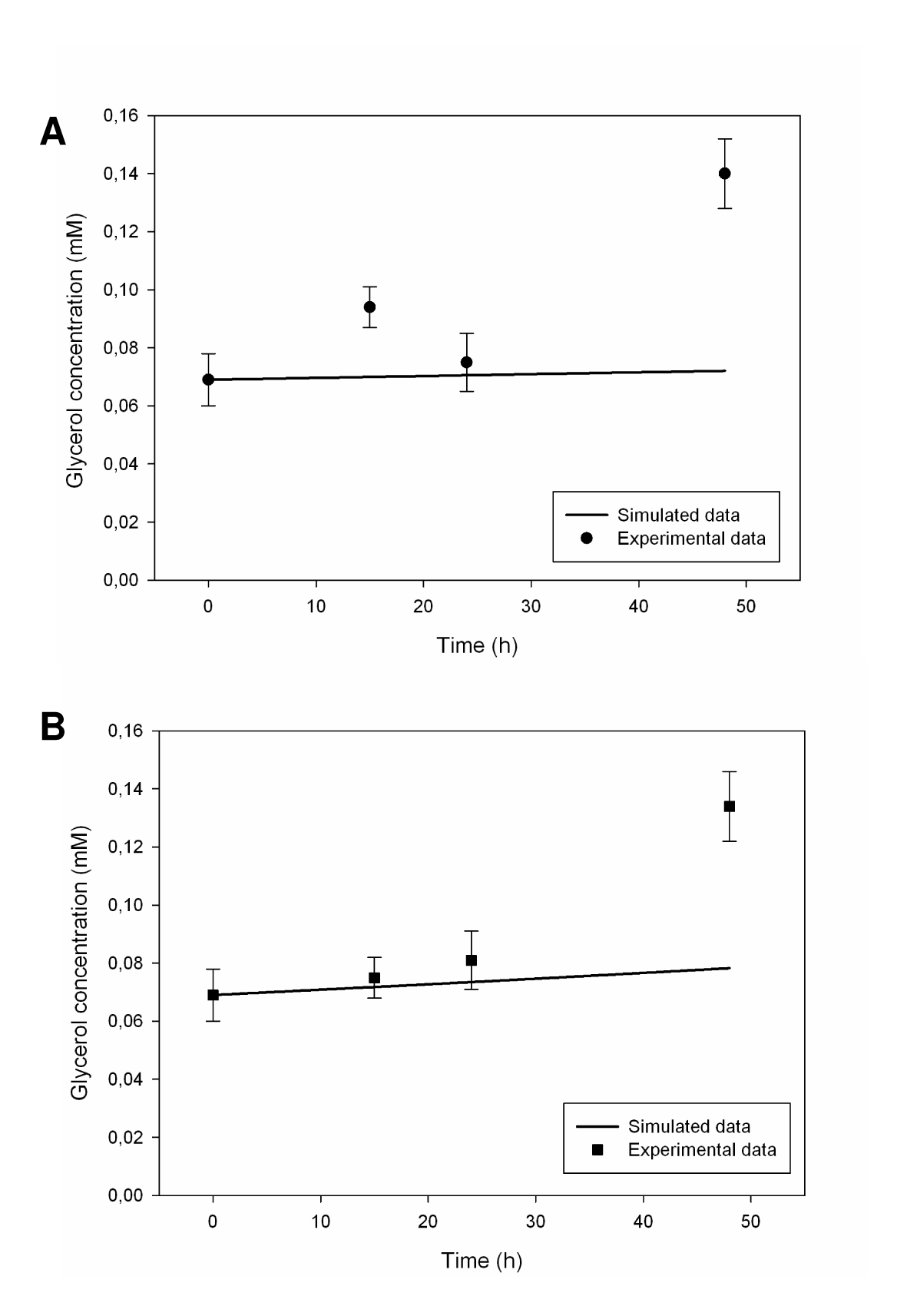

Supplement: S3 Figure — Measured [23] and simulated glycerol trends in the culture medium for adipose monocultures. Upper figure refers to static conditions, the other one describes dynamic conditions. Solid line represents the simulated data, while circles (for the static case) and squares (for the dynamic case) represent the corresponding experimental data. Measured values are expressed as means ± standard deviation for experiments run at least in triplicate: numerical values are reported in [23] and error bars represent the standard deviation. (A) Glycerol trend in static conditions. (B) Glycerol trend in dynamic conditions. (TIF) [file pone.0111946.s003.tif]
