# Supplementary material for: In Silico Models for Dynamic Connected Cell Cultures Mimicking Hepatocyte-Endothelial Cell-Adipocyte Interaction Circle
Source: PLoS One. 2014 Dec 15;9(12):e111946. doi: 10.1371/journal.pone.0111946 (PMC4266517; doi:10.1371/journal.pone.0111946)
Supplement: S1 Table — The full list of all enzymes and consumption terms used in the modelling with their corresponding equations. In reaction kinetics (both in equilibrium and non-equlibrium conditions), oxidated-form cofactors (NAD+, FAD, ADP, NADP+) are considered in saturation, then they not play an active role on the regulation of catalysis rates. It is a good approximation, in fact they are in large quantity in cells. Enzymatic parameters: for direct and indirect reactions, for inhibitors, for direct and indirect reactions. (DOCX) [file pone.0111946.s004.docx]

**Table S1: The full list of all enzymes and consumption terms used in the modelling with their corresponding equations.**

| **Metabolic Pathway**  **and Enzyme** | **Model parameter** | **EC number** | **Kinetic rate equation^1^** |
| --- | --- | --- | --- |
|  |  |  |  |
| **Glycolysis** |  |  |  |
| Glucokinase | GK | 2.7.1.2 | $GK=\frac{V_{\max_{GK}}[ATP][Glu]}{K_{m_{GK}}+[ATP][Glu]}$ |
| Hexokinase | HK | 2.7.1.1 |  |
| Glucose-6-phosphate Isomerase | GPI | 5.3.1.9 |  |
| Phosphofructokinase | PFK | 2.7.1.11 | $PFK=\frac{V_{\max_{PFK}}[ATP][F6P]}{K_{m_{PFK}}+[ATP][F6P]}$ |
| Aldolase | Ald | 4.1.2.13 | $Ald=\frac{V_{\max_{Ald}}(\frac{[FBP]}{K_{m_{Ald_{D}}}}-\frac{[GAP][DHAP]}{K_{m_{Ald_{I}}}})}{1+\frac{[FBP]}{K_{m_{Ald_{D}}}}+\frac{[GAP][DHAP]}{K_{m_{Ald_{I}}}}}$ |
| Triose-phosphate Isomerase | TIM | 5.3.1.1 | $TIM=\frac{V_{\max_{TIM}}(\frac{[DHAP]}{K_{m_{TIM_{D}}}}-\frac{[GAP]}{K_{m_{TIM_{I}}}})}{1+\frac{[DHAP]}{K_{m_{TIM_{D}}}}+\frac{[GAP]}{K_{m_{TIM_{I}}}}}$ |
| GADP Dehydrogenase | GAPDH | 1.2.1.12 | $GAPDH=\frac{V_{\max_{GAPDH}}(\frac{[GAP]}{K_{m_{GAPDH_{D}}}}-\frac{[BPG][NADH]}{K_{m_{GAPDH_{I}}}})}{1+\frac{[GAP]}{K_{m_{GAPDH_{D}}}}+\frac{[BPG][NADH]}{K_{m_{GAPDH_{I}}}}}$ |
| Phosphoglycerate Kinase | PGK | 2.7.2.3 | $PGK=\frac{V_{\max_{PGK}}(\frac{[BPG]}{K_{m_{PGK_{D}}}}-\frac{[3PG][ATP]}{K_{m_{PGK_{I}}}})}{1+\frac{[GAP]}{K_{m_{PGK_{D}}}}+\frac{[3PG][ATP]}{K_{m_{PGK_{I}}}}}$ |
| Phosphoglycerate Mutase | PGM | 5.4.2.1 | $PGM=\frac{V_{\max_{PGM}}(\frac{[3PG]}{K_{m_{PGM_{D}}}}-\frac{[2PG]}{K_{m_{PGM_{I}}}})}{1+\frac{[3PG]}{K_{m_{PGM_{D}}}}+\frac{[2PG]}{K_{m_{PGM_{I}}}}}$ |
| Enolase | Enol | 4.2.1.11 | $Enol=\frac{V_{\max_{Enol}}(\frac{[2PG]}{K_{m_{Enol_{D}}}}-\frac{[PEP]}{K_{m_{Enol_{I}}}})}{1+\frac{[2PG]}{K_{m_{Enol_{D}}}}+\frac{[PEP]}{K_{m_{Enol_{I}}}}}$ |
| Pyruvate Kinase | PK | 2.7.1.40 | $PK=\frac{V_{\max_{PK}}[PEP]}{K_{m_{PK}}+[PEP]}$ |
|  |  |  |  |
| **Gluconeogenesis** |  |  |  |
| Pyruvate carboxylase | PYC | 6.4.1.1 | $PYC=\frac{V_{\max_{PYC}}\left[Pyruv\right]\left[ATP\right]}{K_{m_{PYC}}+\left[Pyruv\right]\left[ATP\right]}$ |
| Phosphoenolpyruvate carboxykinase | PEPCK-C | 4.1.1.32 | $PEPCK-C=\frac{V_{\max_{PEPCK-C}}\left(\frac{\left[Oxalacetate\right]\left[ATP\right]}{K_{m_{PEPCK-C_{D}}}}-\frac{\left[PEP\right]}{K_{m_{PEPCK-C_{I}}}}\right)}{\left(1+\frac{\left[Oxalacetate\right]\left[ATP\right]}{K_{m_{PEPCK-C_{D}}}}+\frac{\left[PEP\right]}{K_{m_{PEPCK-C_{I}}}}\right)}$ |
| Fructose-1,6-bisphosphatase | FBPase | 3.1.3.11 | $FBPase=\frac{V_{\max_{FBPase}}\left[FBP\right]}{K_{m_{FBPase}}+\left[FBP\right]}$ |
| Glucose-6-phosphatase | G6P-C | 3.1.3.9 | $G6P-C=\frac{V_{\max_{G6P-C}}\left(\frac{\left[G6P\right]}{K_{m_{G6P-C_{D}}}}-\frac{\left[Glu\right]}{K_{m_{G6P-C_{I}}}}\right)}{\left(1+\frac{\left[G6P\right]}{K_{m_{G6P-C_{D}}}}+\frac{\left[Glu\right]}{K_{m_{G6P-C_{I}}}}\right)}$ |
|  |  |  |  |
| **Glycogen Biosynthesis** |  |  |  |
| Phosphoglucomutase | PGM | 5.4.2.2 | $PGM=\frac{V_{\max_{PGM}}(\frac{[G6P]}{K_{m_{PGM_{D}}}}-\frac{[G1P]}{K_{m_{PGM_{I}}}})}{1+\frac{[G6P]}{K_{m_{PGM_{D}}}}+\frac{[G1P]}{K_{m_{PGM_{I}}}}}$ |
| UDPG Pyrophosphorilase | UDPGPyr | 2.7.7.9 | $UDPGPyr=\frac{V_{\max_{UDPGPyr}}[G1P][ATP]}{K_{m_{UDPGPyr}}+[G1P][ATP]}$ |
| Glycogen Synthase | GS | 2.4.1.11 | $GS=\frac{V_{\max_{GS}}[UDPG]}{K_{m_{GS}}+[UDPG]}$ |
|  |  |  |  |
| **Glycogen Degradation** |  |  |  |
| Glycogen phosphorylase | GPase | 2.4.1.1 | $GPase=\frac{V_{\max_{GPase}}\left(\frac{\left[Glycogen\right]}{K_{m_{GPase_{D}}}}-\frac{\left[G1P\right]}{K_{m_{GPase_{I}}}}\right)}{\left(1+\frac{\left[Glycogen\right]}{K_{m_{GPase_{D}}}}+\frac{\left[G1P\right]}{K_{m_{GPase_{I}}}}\right)}$ |
| Alpha-1,6-glucosidase | A16G | 3.2.1.33 | $A16G=\frac{V_{\max_{A16G}}\left[Glycogen\right]}{K_{m_{A16G}}+\left[Glycogen\right]}$ |
|  |  |  |  |
| **Glycerol Metabolism** |  |  |  |
| Glycerol kinase | GroK | 2.7.1.30 | $GroK=\frac{V_{\max_{GroK}}\left[Glycerol\right]\left[ATP\right]}{K_{m_{GroK}}+\left[Glycerol\right]\left[ATP\right]}$ |
| Glycerol-3-phosphate dehydrogenase | G3PDH | 1.1.1.8 | $G3PDH=\frac{V_{\max_{G3PDH}}\left(\frac{\left[DHAP\right]\left[NADH\right]}{K_{m_{G3PDH_{D}}}}-\frac{\left[Glycerol3P\right]}{K_{m_{G3PDH_{I}}}}\right)}{\left(1+\frac{\left[DHAP\right]\left[NADH\right]}{K_{m_{G3PDH_{D}}}}+\frac{\left[Glycerol3P\right]}{K_{m_{G3PDH_{I}}}}\right)}$ |
|  |  |  |  |
| **Intermediate Pathways** |  |  |  |
| Malate dehydrogenase | ME | 1.1.1.40 | $ME=\frac{V_{\max_{ME}}\left[Malate\right]}{K_{m_{ME}}+\left[Malate\right]}$ |
| ATP citrate synthase | ACL | 2.3.3.8 | $ACL=\frac{V_{\max_{ACL}}\left[ATP\right]\left[Citrate\right]}{K_{m_{ACL}}+\left[ATP\right]\left[Citrate\right]}$ |
| Pyruvate Dehydrogenase | PDH | 1.2.4.1 | $PDH=\frac{V_{\max_{PDH}}[Pyruv]}{K_{m_{PDH}}+[Pyruv]}$ |
|  |  |  |  |
| **Pentose Phosphate Pathway** |  |  |  |
| G6P Dehydrogenase | G6PD | 1.1.1.49 | $G6PD=\frac{V_{\max_{G6PD}}[G6P]}{K_{m_{G6PD}}(\frac{[NADPH]}{K_{i_{G6PD}}}+1)+[G6P]}$ |
| 6-phosphogluconolactonase | 6PGnL | 3.1.1.31 | $6PGnL=\frac{V_{\max_{6PGnL}}[DGL6P]}{K_{m_{6PGnL}}+[DGL6P]}$ |
| 6PGu dehydrogenase | 6PGuDH | 1.1.1.44 | $6PGuDH=\frac{V_{\max_{6PGuDH}}[6PGu]}{K_{m_{6PGuDH}}(\frac{[NADPH]}{K_{_{6PGuDH}}}+1)+[6PGu]}$ |
| Ribose-5-phosphate isomerase | R5I | 5.3.1.6 | $R5I=\frac{V_{\max_{R5I}}(\frac{[Ru5P]}{K_{m_{R5I_{D}}}}-\frac{[R5P]}{K_{m_{R5I_{I}}}})}{1+\frac{[Ru5P]}{K_{m_{R5I_{D}}}}+\frac{[R5P]}{K_{m_{R5I_{I}}}}}$ |
| Ru5P epimerase | Ru5PE | 5.1.3.1 | $Ru5PE=\frac{V_{\max_{Ru5PE}}(\frac{[Ru5P]}{K_{m_{Ru5PE_{D}}}}-\frac{[Xu5P]}{K_{m_{Ru5PE_{I}}}})}{1+\frac{[Ru5P]}{K_{m_{Ru5PE_{D}}}}+\frac{[Xu5P]}{K_{m_{Ru5PE_{I}}}}}$ |
| Transaldolase | TAL | 2.2.1.2 | $TAL=\frac{V_{\max_{TAL}}(\frac{[GAP][S7P]}{K_{m_{TAL_{D}}}}-\frac{[E4P][F6P]}{K_{m_{TAL_{I}}}})}{1+\frac{[GAP][S7P]}{K_{m_{TAL_{D}}}}+\frac{[E4P][F6P]}{K_{m_{TAL_{I}}}}}$ |
| Transketolase-1 | TKL1 | 2.2.1.1 | $TKL1=\frac{V_{\max_{TKL1}}(\frac{[R5P][Xu5P]}{K_{m_{TKL1_{D}}}}-\frac{[GAP][S7P]}{K_{m_{TKL1_{I}}}})}{1+\frac{[R5P][Xu5P]}{K_{m_{TKL1_{D}}}}+\frac{[GAP][S7P]}{K_{m_{TKL1_{I}}}}}$ |
| Transketolase-2 | TKL2 | 2.2.1.1 | $TKL2=\frac{V_{\max_{TKL2}}(\frac{[E4P][Xu5P]}{K_{m_{TKL2_{D}}}}-\frac{[GAP][F6P]}{K_{m_{TKL2_{I}}}})}{1+\frac{[E4P][Xu5P]}{K_{m_{TKL2_{D}}}}+\frac{[GAP][F6P]}{K_{m_{TKL2_{I}}}}}$ |
|  |  |  |  |
| **Krebs Cycle** |  |  |  |
| Citrate Synthase | CS | 2.3.3.1 | $CS=\frac{V_{\max_{CS}}[Oxaloacetate][AcetylCoA]}{K_{m_{CS}}+[Oxaloacetate][AcetylCoA]}$ |
| Aconitase | Aco | 4.2.1.3 | $Aco=\frac{V_{\max_{Aco}}(\frac{[Citrate]}{K_{m_{Aco_{D}}}}-\frac{[Isocitrate]}{K_{m_{Aco_{I}}}})}{1+\frac{[Citrate]}{K_{m_{Aco_{D}}}}+\frac{[Isocitrate]}{K_{m_{Aco_{I}}}}}$ |
| Isocitrate Dehydrogenase | IsD | 1.1.1.41 | $IsD=\frac{V_{\max_{IsD}}[Isocitrate]}{K_{m_{IsD}}+[Isocitrate]}$ |
| AKG Dehydrogenase | AKGDH | 1.2.7.3 | $AKGDH=\frac{V_{\max_{AKGDH}}[AKG]}{K_{m_{AKGDH}}+[AKG]}$ |
| Succinyl-CoA Synthetase | SCoAS | 6.2.1.4 | $SCoAS=\frac{V_{\max_{SCoAS}}(\frac{[SuccinylCoA]}{K_{m_{SCoAS_{D}}}}-\frac{[Succinate][ATP]}{K_{m_{SCoAS_{I}}}})}{1+\frac{[SuccinylCoA]}{K_{m_{SCoAS_{D}}}}+\frac{[Succinate][ATP]}{K_{m_{SCoAS_{I}}}}}$ |
| Succinate Dehydrogenase | SucDH | 1.3.99.1 | $SucDH=\frac{V_{\max_{SucDH}}(\frac{[Succinate]}{K_{m_{SucDH_{D}}}}-\frac{[Fumarate][FADH_{2}]}{K_{m_{SucDH_{I}}}})}{1+\frac{[Succinate]}{K_{m_{SucDH_{D}}}}+\frac{[Fumarate][FADH_{2}]}{K_{m_{SucDH_{I}}}}}$ |
| Fumarase | Fums | 4.2.1.2 | $Fums=\frac{V_{\max_{Fums}}(\frac{[Fumarate]}{K_{m_{Fums_{D}}}}-\frac{[LMalate]}{K_{m_{Fums_{I}}}})}{1+\frac{[Fumarate]}{K_{m_{Fums_{D}}}}+\frac{[LMalate]}{K_{m_{Fums_{I}}}}}$ |
| Malate Dehydrogenase | LMDH | 1.1.1.37 |  |
|  |  |  |  |
| **Aminoacid Degradation** |  |  |  |
| Alanine Transaminase | AlaT | 2.6.1.2 |  |
| Asparagine Degradation | DAsparag |  | $DAsparag=CAsparag[Asparag]$ |
| Aspartate Transaminase | AspT | 2.6.1.1 | $AspT=\frac{V_{\max_{AspT}}(\frac{[Aspart][AKG]}{K_{m_{AspT_{D}}}}-\frac{[Glut][Oxaloacetate]}{K_{m_{AspT_{I}}}})}{1+\frac{[Aspart][AKG]}{K_{m_{AspT_{D}}}}+\frac{[Glut][Oxaloacetate]}{K_{m_{AspT_{I}}}}}$ |
| Cysteine Degradation | DCys |  | $DCys=CCys[Cys]$ |
| Phenylalanine Degradation | DPhen |  | $DPhen=CPhen[Phen]$ |
| Glycine Degradation | DGly |  | $DGly=CGly[Gly]$ |
| Ornithine Transaminase | OrnT | 2.6.1.13 | $OrnT=\frac{V_{\max_{OrnT}}(\frac{[Orn][AKG]}{K_{m_{OrnT_{D}}}}-\frac{[Glut][Glut5S]}{K_{m_{OrnT_{I}}}})}{1+\frac{[Orn][AKG]}{K_{m_{OrnT_{D}}}}+\frac{[Glut][Glut5S]}{K_{m_{OrnT_{I}}}}}$ |
| Glut5S Dehydrogenase | Glut5SDH | 1.2.1.41 | $Glut5SDH=\frac{V_{\max_{Glut5SDH}}(\frac{[Glut5S]}{K_{m_{Glut5SDH_{D}}}}-\frac{[Glut][NADPH]}{K_{m_{Glut5SDH_{I}}}})}{1+\frac{[Glut5S]}{K_{m_{Glut5SDH_{D}}}}+\frac{[Glut][NADPH]}{K_{m_{Glut5SDH_{I}}}}}$ |
| Glut Dehydrogenase | GlutDH | 1.4.1.2 | $GlutDH=\frac{V_{\max_{GlutDH}}(\frac{[Glut]}{K_{m_{GlutDH_{D}}}}-\frac{[NADPH][NH_{3}][AKG]}{K_{m_{GlutDH_{I}}}})}{1+\frac{[Glut]}{K_{m_{GlutDH_{D}}}}+\frac{[NADPH][NH_{3}][AKG]}{K_{m_{GlutDH_{I}}}}}$ |
| Glutamine Degradation | DGluam |  | $DGluam=CGluam[Gluam]$ |
| Isoleucine Degradation | DIsoleu |  | $DIsoleu=CIsoleu[Gluam][AKG]$ |
| Histidine Degradation | DHist |  | $DHist=CHist[Hist]$ |
| Leucine Degradation | DLeu |  | $DLeu=CLeu[Leu][AKG]$ |
| Lysine Degradation | DLys$DVal=CVal[Val][AKG]$ |  | $DLys=CLys[Lys][AKG][NADPH][NADH]$ |
| Methionine Degradation | DMet |  | $DMet=CMet[Met][ATP][Ser]$ |
| Proline Degradation | DPro |  | $D\Pr o=C\Pr o[\Pr o]$ |
| Serine Degradation | DSer |  | $DSer=CSer[Ser]$ |
| Tyrosine Degradation | DTyr |  | $DTyr=CTyr[Tyr][AKG]$ |
| Threonine Degradation | DThreo |  | $DThreo=CThreo[Threo]$ |
| Tryptophane Degradation | DTryp |  | $DTryp=CTryp[Tryp][NADPH]$ |
| Valine Degradation | DVal |  |  |
| AlphaKetoAdipate Degradation | DAKA |  | $DAKA=CAKA[AKA]$ |
| Propyonil-CoA Degradation | DPropCoA |  | $D\Pr opCoA=C\Pr opCoA[\Pr opCoA][ATP]$ |
|  |  |  |  |
| **Urea Cycle** |  |  |  |
| ArgSucc Synthetase | ArgSucSin | 6.3.4.5 | $ArgSuc\mathrm{Sin}=\frac{V_{\max_{ArgSuc\mathrm{Sin}}}(\frac{[Aspart][Citru][ATP]}{K_{m_{ArgSuc\mathrm{Sin}_{D}}}}-\frac{[ArgSucc]}{K_{m_{ArgSuc\mathrm{Sin}_{I}}}})}{1+\frac{[Aspart][Citru][ATP]}{K_{m_{ArgSuc\mathrm{Sin}_{D}}}}+\frac{[ArgSucc]}{K_{m_{ArgSuc\mathrm{Sin}_{I}}}}}$ |
| ArgininoSuccinase | ArgS | 4.3.2.1 | $ArgS=\frac{V_{\max_{ArgS}}(\frac{[ArgSucc]}{K_{m_{ArgS_{D}}}}-\frac{[Arginine][Fumarate]}{K_{m_{ArgS_{I}}}})}{1+\frac{[ArgSucc]}{K_{m_{ArgS_{D}}}}+\frac{[Arginine][Fumarate]}{K_{m_{ArgS_{I}}}}}$ |
| Arginase | Arginase | 3.5.3.1 | $Arginase=\frac{V_{\max_{Arginase}}[Arginine]}{K_{m_{Arginase}}+[Arginine]}$ |
| Ornithine transcarbamylase | OTransC | 2.1.3.3 | $OTransC=\frac{V_{\max_{OTransC}}(\frac{[Orn][CarbP]}{K_{m_{OTransC_{D}}}}-\frac{[Citru]}{K_{m_{OTransC_{I}}}})}{1+\frac{[Orn][CarbP]}{K_{m_{OTransC_{D}}}}+\frac{[Citru]}{K_{m_{OTransC_{I}}}}}$ |
| CarbP Synthetase | CarbPS | 6.3.4.13 | $CarbPS=\frac{V_{\max_{CarbPS}}[NH_{3}][ATP]^{2}}{K_{m_{CarbPS}}+[NH_{3}][ATP]^{2}}$ |
|  |  |  |  |
| **Fatty acid biosynthesis** |  |  |  |
| Acetyl-CoA carboxylase | ACC | 6.4.1.2 | $ACC=\frac{V_{\max_{ACC}}\left[AcetylCoA\right]\left[ATP\right]}{K_{m_{ACC}}+\left[AcetylCoA\right]\left[ATP\right]}$ |
| Fatty-acid synthase | FAS | 2.3.1.85 | $FAS=\frac{V_{\max_{FAS}}\left[MalonylCoA\right]\left[NADPH\right]}{K_{m_{FAS}}+\left[MalonylCoA\right]\left[NADPH\right]}$ |
|  |  |  |  |
| **Triglyceride Synthesis** |  |  |  |
| Acyl-CoA Synthase | ACAS | 6.2.1.3 | $ACAS=\frac{V_{\max_{ACAS}}\left[FA_{EP}\right]\left[ATP\right]}{K_{m_{ACAS}}+\left[FA_{EP}\right]\left[ATP\right]}$ |
| Glycerol-3-phosphate acyltransferase |  | 2.3.1.15 |  |
| 1-acylglycerol-3-phosphate acyltransferase |  | 2.3.1.51 |  |
| Diacylglycerol acyltransferase |  | 2.3.1.20 |  |
| Synthesis of Triglycerides | STG | (cumulative) | $STG=\frac{V_{\max_{STG}}\left[ACoA\right]\left[Glycerol3P\right]}{K_{m_{STG}}+\left[ACoA\right]\left[Glycerol3P\right]}$ |
|  |  |  |  |
| **Extracellular Lipolysis** |  |  |  |
| Lipoprotein lipase | LPL | 3.1.1.34 | $LPL=\frac{V_{\max_{LPL}}\left[Triclyceride_{EP}\right]}{K_{m_{LPL}}+\left[Triglyceride_{EP}\right]}$ |
|  |  |  |  |
| **Intracellular Lipolysis** |  |  |  |
| Hormone-Sensitive Lipase | HSL | 3.1.1.3 | $HSL=\frac{V_{\max_{HSL}}\left[STG\right]}{K_{m_{HSL}}+\left[STG\right]}$ |
|  |  |  |  |
| **Metabolite transportation** |  |  |  |
| Hepatic Fatty Acid Translocase | CD36EP |  | $CD36_{EP}=\frac{V_{\max_{CD36_{EP}}}\left[FAEX\right]}{K_{m_{CD36_{EP}}}+\left[FAEX\right]}$ |
| Hendothelial Fatty Acid Translocase | CD36ET |  | $CD36_{ET}=\frac{V_{\max_{CD36_{ET}}}\left[FAEX\right]}{K_{m_{CD36_{ET}}}+\left[FAEX\right]}$ |
| Adipose Fatty Acid Translocase | CD36AD |  | $CD36_{AD}=\frac{V_{\max_{CD36_{AD}}}\left[FAEX\right]}{K_{m_{CD36_{AD}}}+\left[FAEX\right]}$ |
| Aquaglyceroporin 7 | AQP7 |  | $AQP7=-\frac{P_{AQP7}\cdot A_{\sup-AD}\cdot\left(Gro-\left[GroEX\right]\right)}{V_{AD}}$ |
| Aquaglyceroporin 9 | AQP9 |  | $AQP9=-\frac{P_{AQP9}\cdot A_{\sup-EP}\cdot\left(Gro-\left[GroEX\right]\right)}{V_{EP}}$ |

^1^ Abbreviations: ATP (Adenosine TriPhosphate); Pyruv (Pyruvate); PEP (PhosphoEnolPyruvate); FBP (Fructose-BisPhosphate); G6P (Glucose-6-Phosphate); Glu (Glucose); G1P (Glucose-1-Phosphate); CD36_EP,ET,AD_: Fatty Acid Translocase for Hepatic, Endothelial and Adipose Cell, respectively; P_AQP7,AQP9_: membrane permeability due to adipose or hepatic aquaglyceroporins, respectively; A_sup-AD,sup-EP_: mean surface area of an adipocyte or a hepatocyte, respectively; V_AD,EP_: mean volume of an adipocyte or a hepatocyte, respectively; *etc.* (for the other abbreviations, please see Table SM 2).
