# Supplementary material for: In Silico Models for Dynamic Connected Cell Cultures Mimicking Hepatocyte-Endothelial Cell-Adipocyte Interaction Circle
Source: PLoS One. 2014 Dec 15;9(12):e111946. doi: 10.1371/journal.pone.0111946 (PMC4266517; doi:10.1371/journal.pone.0111946)
Supplement: S2 Table — The full list of state equations used in the modelling. (DOCX) [file pone.0111946.s005.docx]

**Table S2: The full list of state equations used in the modelling.**

| **Metabolic Pathway**  **and Metabolite** | **Model parameter** | **State equation^1^** |
| --- | --- | --- |
|  |  |  |
| **Carbohydrate Metabolism** |  |  |
| Hepatic Extracellular Glucose | GluEX | $\frac{d\left[GluEX\right]}{dt}=-C_{EPg}N_{EP}\left[GluEX\right]+R_{EPg}N_{EP}\left[Glu\right]$ |
| Endothelial Extracellular Glucose | GluEX | $\frac{d\left[GluEX\right]}{dt}=-C_{ETg}N_{ET}\left[GluEX\right]+R_{ETg}N_{ET}\left[Glu\right]$ |
| Adipose Extracellular Glucose | GluEX | $\frac{d\left[GluEX\right]}{dt}=-C_{ADg}N_{AD}\left[GluEX\right]+R_{ADg}N_{AD}\left[Glu\right]$ |
| Hepatic Intracellular Glucose | Glu | $\frac{d\left[Glu\right]}{dt}=C_{EPg}\left[GluEX\right]-GK+G6Pc+A16G$ |
| Endothelial Intracellular Glucose | Glu | $\frac{d\left[Glu\right]}{dt}=C_{ETg}\left[GluEX\right]-HK+G6Pc$ |
| Adipose Intracellular Glucose | Glu | $\frac{d\left[Glu\right]}{dt}=C_{ADg}\left[GluEX\right]-HK$ |
| Hepatic Glucose-6-Phosphate | G6P | $\frac{d\left[G6P\right]}{dt}=GK-GPI-PGM-G6PD-G6Pc$ |
| Endothelial Glucose-6-Phosphate | G6P | $\frac{d\left[G6P\right]}{dt}=HK-GPI-PGM-G6PD-G6Pc$ |
| Adipose Glucose-6-Phosphate | G6P | $\frac{d\left[G6P\right]}{dt}=HK-GPI-PGM-G6PD$ |
| Hepatic Fructose-6-Phosphate | F6P | $\frac{d\left[F6P\right]}{dt}=GPI-PFK+TAL+TKL2+FBPase$ |
| Endothelial Fructose-6-Phosphate | F6P | $\frac{d\left[F6P\right]}{dt}=GPI-PFK+TAL+TKL2+FBPase$ |
| Adipose Fructose-6-Phosphate | F6P | $\frac{d[F6P]}{dt}=GPI-PFK+TAL+TKL2$ |
| Hepatic  Fructose-1,6-bisphosphate | FBP | $\frac{d\left[FBP\right]}{dt}=PFK-Ald-FBPase$ |
| Endothelial  Fructose-1,6-bisphosphate | FBP | $\frac{d\left[FBP\right]}{dt}=PFK-Ald-FBPase$ |
| Adipose Fructose-1,6-bisphosphate | FBP | $\frac{d[FBP]}{dt}=PFK-Ald$ |
| Hepatic Dihydroxyacetone  phosphate | DHAP | $\frac{d\left[DHAP\right]}{dt}=Ald-TIM-G3PDH$ |
| Endothelial  Dihydroxyacetone phosphate | DHAP | $\frac{d\left[DHAP\right]}{dt}=Ald-TIM-G3PDH$ |
| Adipose  Dihydroxyacetone phosphate | DHAP | $\frac{d\left[DHAP\right]}{dt}=Ald-TIM-G3PDH$ |
| Glyceraldehyde-3-phosphate | GAP | $\frac{d\left[GAP\right]}{dt}=TIM-TAL+TKL2+TKL1-GAPD+Ald$ |
| 1,3-Bisphospho-glycerate | BPG | $\frac{d[BPG]}{dt}=GAPDH-PGK$ |
| 3-Phospho-glycerate | 3PG | $\frac{d[3PG]}{dt}=PGK-PGM$ |
| 2-Phospho-glycerate | 2PG | $\frac{d[2PG]}{dt}=PGM-Enol$ |
| Hepatic Phosphoenolpyruvate | PEP | $\frac{d\left[PEP\right]}{dt}=Enol-PK+PEPCKc$ |
| Endothelial Phosphoenolpyruvate | PEP | $\frac{d\left[PEP\right]}{dt}=Enol-PK+PEPCKc$ |
| Adipose Phosphoenolpyruvate | PEP | $\frac{d[PEP]}{dt}=Enol-PK$ |
| Hepatic Pyruvate | Pyruv | $\frac{d\left[Pyruv\right]}{dt}=PK-PYC-PDH+AlaT$ |
| Endothelial Pyruvate | Pyruv | $\frac{d\left[Pyruv\right]}{dt}=PK-PYC-PDH+AlaT$ |
| Adipose Pyruvate | Pyruv | $\frac{d[Pyruv]}{dt}=PK-PDH+AlaT$ |
| Hepatic Glycogen | Glycogen | $\frac{d\left[Glycogen\right]}{dt}=GS-GPase-A16G$ |
| Hepatic Uridine Diphosphate  Glucose | UDPG | $\frac{d[UDPG]}{dt}=UDPGPyr-GS$ |
| Hepatic Glucose-1-Phosphate | G1P | $\frac{d\left[G1P\right]}{dt}=PGM-UDPGPyr+GPase$ |
| D-Glucono-1,5-lactone 6-phosphate | DGL6P | $\frac{d[DGL6P]}{dt}=G6PD-6PGnL$ |
| 6-Phospho-gluconate | 6PGu | $\frac{d[6PGu]}{dt}=6PGnL-6PGuDH$ |
| Ribulose-5-phosphate | Ru5 | $\frac{d[Ru5P]}{dt}=6PGuDH-R5I-Ru5PE$ |
| Ribose-5-phosphate | R5P | $\frac{d[R5P]}{dt}=R5I-TKL1-Duplic[R5P]$ |
| Xylulose-5-phosphate | Xu5P | $\frac{d[Xu5P]}{dt}=Ru5PE-TKL1-TKL2$ |
| D-Sedoheptulose-7-phosphate | S7P | $\frac{d[S7P]}{dt}=TKL1-TAL$ |
| Erythrose-4-phosphate | E4P | $\frac{d[E4P]}{dt}=TAL-TKL2$ |
|  |  |  |
| **Krebs Cycle** |  |  |
| Hepatic Oxalacetate | Oxaloacetate | $\frac{d\left[Oxalacetate\right]}{dt}=AspT+LMDH-CS+PYC-PEPCKc$ |
| Endothelial Oxalacetate | Oxaloacetate | $\frac{d\left[Oxalacetate\right]}{dt}=AspT+LMDH-CS+PYC-PEPCKc$ |
| Adipose Oxaloacetate | Oxaloacetate | $\frac{d[Oxaloacetate]}{dt}=AspT+LMDH-CS$ |
| Citrate | Citrate | $\frac{d[Citrate]}{dt}=CS-Aco$ |
| Isocitrate | Isocitrate | $\frac{d[Isocitrate]}{dt}=Aco-IsD$ |
| Alpha-Ketoglutaric acid | AKG | $\begin{matrix} \frac{d[AKG]}{dt}=IsD-AKGDH-DTir-AlaT-AspT-OrnT+ \\ GlutDH-DIsoleu-2\cdot DLys-DLeu-DTir-DVal \end{matrix}$ |
| Succinyl-CoA | Succinyl-CoA | $\frac{d[SuccinylCoA]}{dt}=AKGDH-SCoAS+D\Pr opCoA$ |
| Succinate | Succinate | $\frac{d[Succinate]}{dt}=SCoAS-SucDH$ |
| Fumarate | Fumarate | $\frac{d[Fumarate]}{dt}=SucDH-Fums+DTir+ArgS$ |
| Malate | Malate | $\frac{d[Malate]}{dt}=Fums-LMDH$ |
|  |  |  |
| **Aminoacid Degradation** |  |  |
| Extracellular Generic Aminoacid | aaEX | $\frac{d\left[aaEX\right]}{dt}=-AC\cdot N\cdot\left[aaEX\right]$ |
| Alanine | Ala | $\frac{d[Ala]}{dt}=AC[AlaEX]+DTryp-AlaT$ |
| Arginine | Arginine | $\frac{d[Arginine]}{dt}=AC[ArginineEX]+ArgS-Arginase$ |
| Asparagine |  |  |
| Asparate | Aspart | $\frac{d[Aspart]}{dt}=AC[AspartEX]+DAsparag-ArgSuc\mathrm{Sin}-AspT$ |
| Cysteine | Cys | $\frac{d[Cys]}{dt}=AC[CysEX]+DMet-DCys$ |
| Phenylalanine | Phen | $\frac{d[Phen]}{dt}=AC[PhenEX]-DPhen$ |
| Glycine | Gly | $\frac{d[Gly]}{dt}=AC[GlyEX]+DThreo-DGly$ |
| Glutamate | Glut | $\begin{matrix} \frac{d[Glut]}{dt}=AC[GlutEX]+OrnT+DIst+DGluam+Glut5SDH-Glut5SDH \\ -GlutDH+DIsoleu+DVal+DLeu+DTyr+AspT+2\cdot DLys+AlaT \end{matrix}$ |
| Glutamine | Gluam | $\frac{d[Gluam]}{dt}=AC[GluamEX]-DGluam$ |
| Isoleucine | Isoleu | $\frac{d[Isoleu]}{dt}=AC[IsoleuEX]-DIsoleu$ |
| Histidine | Hist | $\frac{d[Hist]}{dt}=AC[HistEX]-DHist$ |
| Leucine | Leu | $\frac{d[Leu]}{dt}=AC[LeuEX]-DLeu$ |
| Lysine | Lys | $\frac{d[Lys]}{dt}=AC[LysEX]-DLys$ |
| Methionine | Met | $\frac{d[Met]}{dt}=AC[MetEX]-DMet$ |
| Proline | Pro | $\frac{d[\Pr o]}{dt}=AC[\Pr oEX]-D\Pr o$ |
| Serine | Ser | $\frac{d[Ser]}{dt}=AC[SerEX]+\frac{1}{2}DGly-DSer-DMet$ |
| Tyrosine | Tyr | $\frac{d[Tyr]}{dt}=AC[TyrEX]-DTyr+DPhen$ |
| Threonine | Threo | $\frac{d[Threo]}{dt}=AC[ThreoEX]-DThreo$ |
| Tryptophane | Tryp | $\frac{d[Tryp]}{dt}=AC[TrypEX]-DTryp$ |
| Valine | Val | $\frac{d[Val]}{dt}=AC[ValEX]-DVal$ |
| Glutamate 5-semialdehyde | Glut5S | $\frac{d[Glut5S]}{dt}=OrnT+D\Pr o-Glut5SDH$ |
| Propyonil-CoA | PropCoA | $\frac{d[\Pr opCoA]}{dt}=DMet+DVal+DIsoleu-D\Pr opCoA$ |
| AlphaKetoAdipate | AKA | $\frac{d[AKA]}{dt}=DLis+DTryp-DAKA$ |
|  |  |  |
| **Urea Cycle** |  |  |
| Carbamoyl-Phosphate | CarbP | $\frac{d[CarbP]}{dt}=CarbPS-OtransC$ |
| Ornithine | Orn | $\frac{d[Orn]}{dt}=Arginase-OrnT-OtransC$ |
| Citrulline | Citr | $\frac{d[Citr]}{dt}=OtransC-ArgSuc\mathrm{Sin}$ |
| Argininosuccinate | ArgSucc | $\frac{d[ArgSucc]}{dt}=ArgSuc\mathrm{Sin}-ArgS$ |
| Ammonia | NH_3_ | $\begin{matrix} \frac{d[NH_{3}]}{dt}=DCys+DSer+DGly+DHist+DGluam+GlutDH \\ +DAsparag+DMet-CarbPS \end{matrix}$ |
| Urea | Urea | $\frac{d[Urea]}{dt}=Arginase$ |
|  |  |  |
| **Glycerol Metabolism** |  |  |
| Hepatic Extracellular Glycerol | GroEX | $\frac{d\left[GroEX\right]}{dt}=-AQP9\cdot N_{EP}$ |
| Adipose Extracellular Glycerol | GroEX | $\frac{d\left[GroEX\right]}{dt}=+AQP7\cdot N_{AD}$ |
| Hepatic Intracellular Glycerol | Gro | $\frac{d\left[Gro\right]}{dt}=AQP9-GroK$ |
| Adipose Intracellular Glycerol | Gro | $\frac{d\left[Gro\right]}{dt}=HSL-AQP7$ |
| Hepatic Glycerol-3-Phosphate | Gro3P | $\frac{d\left[Gro3P\right]}{dt}=GroK+G3PDH-STG$ |
| Endothelial Glycerol-3-Phosphate | Gro3P | $\frac{d\left[Gro3P\right]}{dt}=G3PDH-STG$ |
| Adipose Glycerol-3-Phosphate | Gro3P | $\frac{d\left[Gro3P\right]}{dt}=G3PDH-STG$ |
|  |  |  |
| **Lipid Metabolism** |  |  |
| Hepatic Acetyl-CoA | Acetyl-CoA | $\frac{d\left[Acetyl-CoA\right]}{dt}=+PDH-ACC-CS+DTir+2DLeu+DTreo+DIsoleu+2DAKA$ |
| Endothelial Acetyl-CoA | Acetyl-CoA | $\frac{d\left[Acetyl-CoA\right]}{dt}=+PDH-ACC-CS+DTir+2DLeu+DTreo+DIsoleu+2DAKA$ |
| Hepatic Malonil-CoA | MCoA | $\frac{d\left[MCoA\right]}{dt}=ACC-FAS$ |
| Endothelial Malonil-CoA | MCoA | $\frac{d\left[MCoA\right]}{dt}=ACC-FAS$ |
| Hepatic Extracellular Fatty Acids | FAEX | $\frac{d\left[FAEX\right]}{dt}=-CD36_{EP}\cdot N_{EP}$ |
| Endothelial Extracellular  Fatty Acids | FAEX | $\frac{d\left[FAEX\right]}{dt}=0,40\cdot3\cdot TG_{ET}\cdot N_{ET}-CD36_{ET}\cdot N_{ET}$ |
| Adipose Extracellular Fatty Acids | FAEX | $\frac{d\left[FAEX\right]}{dt}=-CD36_{AD}\cdot N_{AD}+3\cdot HSL\cdot\left(1-0.25\right)$ |
| Hepatic Intracellular Fatty Acids | FA_EP_ | $\frac{d\left[FA_{EP}\right]}{dt}=FAS-ACAS+CD36_{EP}$ |
| Endothelial Intracellular Fatty Acids | FA_ET_ | $\frac{d\left[FA_{ET}\right]}{dt}=FAS-ACAS+CD36_{ET}$ |
| Adipose Intracellular Fatty Acids | FA_AD_ | $\frac{d\left[FA_{AD}\right]}{dt}=FAS-ACAS+CD36_{AD}$ |
| Adipose Intracellular Fatty Acids for Internal Riesterification | FA_ADr_ | $\frac{d\left[FA_{ADr}\right]}{dt}=0.25\cdot\left(3\cdot HSL\right)$ |
| Hepatic Acyl-CoA | ACoA | $\frac{d\left[ACoA\right]}{dt}=ACAS-STG$ |
| Endothelial Acyl-CoA | ACoA | $\frac{d\left[ACoA\right]}{dt}=ACAS-STG$ |
| Adipose Acyl-CoA | ACoA | $\frac{d\left[ACoA\right]}{dt}=ACAS-STG$ |
| Hepatic Intracellular Triglycerides | TG_EP_ | $\frac{d\left[TG_{EP}\right]}{dt}=STG$ |
| Endothelial Intracellular Triglycerides | TG_ET_ | $\frac{d\left[TG_{ET}\right]}{dt}=STG-0,40\cdot3\cdot TG_{ET}$ |
| Adipose Intracellular Triglycerides | TG_AD_ | $\frac{d\left[TG_{AD}\right]}{dt}=STG-HSL$ |
|  |  |  |
| **Energy Balance** |  |  |
| NADH | NADH | $\frac{d[NADH]}{dt}=\sum\mathrm{mod}\_NADH-C\_NADH[NADH][ATP]$ |
| FADH_2_ | FADH_2_ | $\frac{d[FADH_{2}]}{dt}=\sum\mathrm{mod}\_FADH_{2}-C\_FADH_{2}[FADH_{2}][ATP]$ |
| ATP | ATP | $\frac{d[ATP]}{dt}=\sum\mathrm{mod}\_ATP-C\_ATP[ATP]$ |
| NADPH | NADPH | $\frac{d[NADPH]}{dt}=\sum\mathrm{mod}\_NADPH-C\_NADPH[NADPH]$ |
|  |  |  |
| **Metabolite Concentration**  **employed only for the 3-way Connected Culture System** |  |  |
| Hepatic Intracellular Triglycerides | TG_EP_ | $\frac{d\left[TG_{EP}\right]}{dt}=STG-LPL$ |
| Extracellular Glucose | GluEX | $\begin{matrix} \frac{d\left[GluEX\right]}{dt}=-C_{ETg}N_{ET}\left[GluEX\right]+R_{ETg}N_{ET}\left[Glu_{ET}\right]-C_{ADg}N_{AD}\left[GluEX\right]+R_{ADg}N_{AD}\left[Glu_{AD}\right] \\ -C_{EPg}N_{EP}\left[GluEX\right]+R_{EPg}N_{EP}\left[Glu_{EP}\right] \end{matrix}$ |
| Extracellular Fatty Acids | FAEX | $\begin{matrix} \frac{d\left[FAEX\right]}{dt}=-CD36_{ET}\cdot N_{ET}+0,40\cdot3\cdot TG_{ET}\cdot N_{ET}-CD36_{AD}\cdot N_{AD}+3\cdot HSL\cdot\left(1-0.25\right) \\ -CD36_{EP}\cdot N_{EP}+LPL\cdot N_{EP} \end{matrix}$ |
| Extracellular Glycerol | GroEX | $\frac{d\left[GroEX\right]}{dt}=+AQP7\cdot N_{AD}-AQP9\cdot N_{EP}$ |
|  |  |  |

^1^ Abbreviations: ATP (Adenosine TriPhosphate); NADH (Nicotidamide Adenine Dinucleotide); FADH_2_ (Flavine Adenine Dinucleotide); NADPH (Nicotinamide Adenine Dinucleotide Phosphate); Pyruv (Pyruvate); PEP (PhosphoEnolPyruvate); FBP (Fructose-BisPhosphate); G6P (Glucose-6-Phosphate); Glu (Glucose); G1P (Glucose-1-Phosphate); C_EPg,ETg,ADg_, and R_EPg,ETg,ADg_: glucose uptake and release constants for the hepatocyte, the endothelial cell and the adipocyte, respectively; N_EP,ET,AD_: number of cells for hepatic, endothelial and adipose population, respectively; AC: generic aminoacid uptake constant for all kinds of cells considered; mod_ATP, mod_NADH, mod_FADH_2_, mod_NADPH: terms representing the increment/or consumption of the energy molecules due to the metabolic pathways modelled; C_ATP, C_NADH, C_FADH_2_, C_NADPH: terms representing the consumption of the energy molecules due to the metabolic processes not modelled; *etc.* (for the other abbreviations, please see Table SM 1).
