# Supplementary material for: In Silico Models for Dynamic Connected Cell Cultures Mimicking Hepatocyte-Endothelial Cell-Adipocyte Interaction Circle
Source: PLoS One. 2014 Dec 15;9(12):e111946. doi: 10.1371/journal.pone.0111946 (PMC4266517; doi:10.1371/journal.pone.0111946)
Supplement: S3 Table — The full list of stoichiometric equations used in the modelling for aminoacid degradation. (DOCX) [file pone.0111946.s006.docx]

**Table S3: The full list of stoichiometric equations used in the modelling for aminoacid degradation.**

| **Stoichiometric Equations for Aminoacid Degradation** |
| --- |
| Alanine + AKG → Glutamate + Pyruvate |
| Arginine → Ornitine + Urea |
| Asparagine → Aspartate + NH_3_ |
| Aspartate + AKG → Glutamate + Oxaloacetate |
| Cysteine → NH_3_ + Pyruvate |
| Phenylalanine → Tyrosine |
| 2 Glycine → NH_3_ + NADH + Serine |
| Glutamate → NH_3_ + NADPH + AKG |
| Glutamine → NH_3_ + Glutamate |
| Isoleucine + AKG → NADH + Propyonil-CoA + Acetyl-CoA + Glutamate + FADH_2_ |
| Histidine → NH_3_ + Glutamate |
| Leucine + AKG → NADH + 2 Acetyl-CoA + Glutamate + FADH_2_ |
| 2 Lysine + NADPH + NADH + 4 AKG → 4 Glutamate + AlphaKetoAdipate |
| Methionine + ATP + Serine → NH_3_ + Cysteine + NADH + Propyonil |
| Serine → NH_3_ + Pyruvate |
| Proline → Glutamate 5-semialdehyde |
| Tyrosine + AKG → Glutamate + Fumarate + Acetyl-CoA |
| Threonine → Glycine + Acetyl-CoA |
| Tryptophane + NADPH → Alanine + AlphaKetoAdipate |
| Valine + AKG → NADH + Propyonil-CoA + Glutamate + FADH_2_ |
| Glutamate 5-semialdehyde → NADPH + Glutamate |
| Propyonil-CoA + ATP → Succinyl-CoA |
| AlphaKetoAdipate → 2 Acetyl-CoA + FADH_2_ +NADH |
